# CYP5122A1 encodes an essential sterol C4-methyl oxidase in *Leishmania donovani* and determines the antileishmanial activity of antifungal azoles

**DOI:** 10.21203/rs.3.rs-3185204/v1

**Published:** 2023-07-27

**Authors:** Yiru Jin, Somrita Basu, Mei Feng, Yu Ning, Indeewara Munasinghe, Arline M. Joachim, Junan Li, Robert Madden, Hannah Burks, Philip Gao, Chamani Perera, Karl A. Werbovetz, Kai Zhang, Michael Zhuo Wang

**Affiliations:** 1 Department of Pharmaceutical Chemistry, School of Pharmacy, The University of Kansas, Lawrence, KS 66047, USA; 2 Division of Medicinal Chemistry and Pharmacognosy, College of Pharmacy, The Ohio State University, Columbus, OH 43210, USA; 3 College of Pharmacy, The Ohio State University, Columbus, Ohio 43210, USA; 4 Department of Biological Sciences, Texas Tech University, Lubbock, TX 79409, USA; 5 Synthetic Chemical Biology Core Laboratory, The University of Kansas, Lawrence, KS 66047, USA; 6 Protein Production Group, The University of Kansas, Lawrence, KS 66047, USA

## Abstract

Visceral leishmaniasis, caused by *Leishmania donovani*, is a life-threatening parasitic disease, but current antileishmanial drugs are limited and have severe drawbacks. There have been efforts to repurpose antifungal azole drugs for the treatment of *Leishmania* infection. Antifungal azoles are known to potently inhibit the activity of cytochrome P450 (CYP) 51 enzymes which are responsible for removing the C14α-methyl group of lanosterol, a key step in ergosterol biosynthesis in *Leishmania*. However, they exhibit varying degrees of antileishmanial activities in culture, suggesting the existence of unrecognized molecular targets for these compounds. Our previous study reveals that, in *Leishmania*, lanosterol undergoes parallel C4- and C14-demethylation reactions to form 4α,14α-dimethylzymosterol and T-MAS, respectively. In the current study, CYP5122A1 is identified as a sterol C4-methyl oxidase that catalyzes the sequential oxidation of lanosterol to form C4-oxidation metabolites. CYP5122A1 is essential for both *L. donovani* promastigotes in culture and intracellular amastigotes in infected mice. Overexpression of CYP5122A1 results in growth delay, differentiation defects, increased tolerance to stress, and altered expression of lipophosphoglycan and proteophosphoglycan. CYP5122A1 also helps to determine the antileishmanial effect of antifungal azoles *in vitro*. Dual inhibitors of CYP51 and CYP5122A1, e.g., clotrimazole and posaconazole, possess superior antileishmanial activity against *L. donovani* promastigotes whereas CYP51-selective inhibitors, e.g., fluconazole and voriconazole, have little effect on promastigote growth. Our findings uncover the critical biochemical and biological role of CYP5122A1 in *L. donovani* and provide an important foundation for developing new antileishmanial drugs by targeting both CYP enzymes.

## Introduction

Human leishmaniasis is a complex of diseases caused by protozoan parasites of the genus *Leishmania*. The *Leishmania* parasites exist in the form of replicative promastigotes in the midgut of female sand flies. During a sand fly’s blood meal, the infective metacyclic promastigotes are injected into the host and then taken up by macrophages, where they transform into intracellular amastigotes that can survive, persist, and disseminate within the host [1–3]. The main clinical manifestations include cutaneous, mucocutaneous, and visceral leishmaniasis. Among them, visceral leishmaniasis (VL) is the most severe, affecting liver, spleen, and bone marrow, and is fatal in over 95% of cases if left untreated [4]. There are an estimated 50000 to 90000 new VL cases per year globally [4]. VL is caused by *Leishmania donovani* in East Africa and on the Indian subcontinent and by *Leishmania infantum* in Europe, North Africa, and Latin America [5].

To date, there is no human vaccine for VL [6]. Chemotherapeutic drugs are limited and have drawbacks such as toxicity, long treatment regimens, emerging resistance, and/or high cost [7]. Therefore, there is still an unmet medical need for safe and effective antileishmanial drugs. The sterol biosynthesis pathway in *Leishmania* has been reported as a potential antileishmanial drug target [8, 9]. Sterols (e.g., cholesterol and ergosterol) are integral components of biological membranes for regulating membrane fluidity and permeability. In addition, studies have shown that the perturbation of the sterol composition in *Leishmania* spp. led to mitochondrion dysfunction, superoxide accumulation, hypersensitivity to heat, and/or attenuated virulence in mice [10–12]. Unlike mammalian cells that synthesize cholesterol, *Leishmania* parasites synthesize ergosterol and other ergostane-based sterols. Ergosterol biosynthesis begins with the removal of methyl groups at lanosterol C4 and C14 positions (Scheme 1). The C14-demethylation of the sterol precursor lanosterol is mediated by the sterol C14α-demethylase (CYP51). Leishmanial CYP51 can be inhibited by antifungal azole drugs, a class of fungal CYP51 inhibitors, which results in the accumulation of C14-methylated sterol intermediates and the depletion of ergostane-based sterols [13]. However, antifungal azoles exhibited significantly different antileishmanial activities *in vitro*. For example, ketoconazole or itraconazole at 1 μg/mL inhibited the growth of multiple strains of *L. donovani* and *L. infantum* promastigotes (<20% growth compared with control) whereas fluconazole did not (>80% growth) [13]. Fenticonazole and tioconazole were able to eliminate the intracellular amastigotes of *L. infantum* with EC_50_ values of 2.9 ± 0.3 μM and 4.0 ± 0.2 μM but voriconazole failed [14]. Miconazole and clotrimazole were submicromolar inhibitors of intracellular *L. major* and *L. amazonensis*, while fluconazole and voriconazole were ineffective against these intracellular parasites (<50% inhibition) at 10 μM concentrations [15]. These results suggest that there may be other molecular targets in play that determine the antileishmanial activity of antifungal azoles. Our previous study examined differential sterol profiles of azole-treated *L. donovani* promastigotes and proposed a branched ergosterol biosynthetic pathway in *Leishmania* [16]. Specifically, the C14- and C4-demethylation reactions of lanosterol occur in parallel rather than sequentially as in fungi and the inhibition of both reactions may be required for optimal antileishmanial effect. As such, identification and characterization of the enzyme(s) responsible for lanosterol C4-demethylation is of paramount importance to understand sterol biology in *Leishmania* and lay a foundation to discover effective antileishmanial treatments that target the sterol biosynthetic pathway.

In most eukaryotes, the O_2_-dependent sterol C4 demethylation is catalyzed by three enzymes: a C4 sterol methyl oxidase that sequentially generates hydroxy, aldehyde and carboxylate intermediates, a C4 decarboxylase, and a 3-ketosterol reductase (ERG27) [17–19]. In yeast and vertebrates, the same C4 sterol methyl oxidase (ERG25) works on both C4 methyl groups, whereas plants have two distinct ERG25 homologs that oxidize 4,4-dimethylsterols and 4α-methylsterols, respectively [20]. To date, information regarding the identity and therapeutic implications of sterol C4-demethylase in *Leishmania* remains scarce, which is in sharp contrast to CYP51 which has been investigated biochemically, genetically and pharmacologically in *Leishmania* and related trypanosomatids [21–24].

CYP5122A1 is a recently identified cytochrome P450 (CYP) enzyme which is well conserved among trypanosomatids [25] (Supplementary Fig. 1). Its amino acid sequence is only 22% identical to the CYP51 sequence and hence they belong to different CYP families, i.e., family 5122 and 51, respectively. It was reported that a heterozygous deletion of the *CYP5122A1* gene impairs parasite growth, mitochondrial function, and infectivity, although a *CYP5122A1* null mutant was untenable [25]. In addition, it was suggested that CYP5122A1 could be involved in ergosterol biosynthesis in *Leishmania* as the ergosterol content was reduced by 70% in the heterozygous knockout parasites and partially restored by complementation of *CYP5122A1* through episomal expression [25]. Moreover, CYP5122A1 heterozygous knockout parasites were significantly more sensitive to ketoconazole than the wild-type *L. donovani* promastigotes, although the underlying mechanism remains unknown [26]. It is also not clear if CYP5122A1 catalyzes the C4-demethylation reaction as CYP51 does for the C14-demethylation.

In this study, an N-terminal truncated construct of *L. donovani* CYP5122A1 was cloned, heterologously expressed, purified, and spectrally characterized. By reconstituting the catalytic activity of CYP5122A1 *in vitro*, its biochemical function in ergosterol biosynthesis was elucidated. Additionally, the essentiality of CYP5122A1 was evaluated for both *L. donovani* promastigotes and amastigotes using a complementing episome-assisted knockout approach coupled with negative selection. The effects of CYP5122A1 on cell growth, differentiation, stress responses, and expression of surface glycoconjugates were also assessed. Lastly, the association between CYP5122A1 inhibition and antileishmanial activities of antifungal azoles was determined. These results support that CYP5122A1 acts as the bona fide sterol C4-methyl oxidase, is essential to *L. donovani*, and determines the antileishmanial activity of antifungal azoles.

## Materials and Methods

### Chemicals and Reagents

Lanosterol (>=93% pure) and reduced nicotinamide adenine dinucleotide phosphate (NADPH) were obtained from Sigma-Aldrich (St. Louis, MO). 5α-Cholesta-8,24-dien-3β-ol (zymosterol), 14-demethyl-14-dehydrolanosterol (FF-MAS), 4,4-dimethylcholesta-8,24-dien-3β-ol (T-MAS), and cholesterol-d7 were purchased from Avanti Polar Lipids Inc (Alabaster, AL). 4α,14α-Dimethylzymosterol (4,14-DMZ) was isolated from the sterol extract of *L. tarentolae* [16]. Dilaurylphosphatidylcholine (DLPC), dimyristoylphosphatidylcholine (DMPC), and dimyristoylphosphatidylglycerol (DMPG) were a gift from Dr. Philip Gao at the Protein Production Group, University of Kansas. Emulgen 911 was purchased from Desert Biologicals (Phoenix, AZ). Reagents used in *Leishmania* work were purchased from Thermo Fisher Scientific (Waltham, MA) or VWR (Radnor, PA) unless otherwise specified.

### Cloning, expression, and purification

The CYP5122A1 and CYP51 enzymes used in this study were the truncated forms of the full-length proteins (XP_003861867.1 and XP_003859085.1) without the transmembrane domains (the first 60 and 31 amino acids for CYP5122A1 and CYP51 as shown in Supplementary Fig. 1). A solubility tag MAKKTSSKGKL [27] and a His-tag were added to the N- and C-terminus, respectively, to facilitate protein purification. Their gene sequences were incorporated into the pCWori vector (a gift from Dr. Emily Scott; University of Michigan) and confirmed by Sanger sequencing prior to expression. NEB^®^ 5-alpha F’*Iq* competent *E. coli* (New England Biolabs) transformed with the plasmids were grown at 37 °C. When OD_600_ reached 0.6, isopropyl β-D-1-thiogalactopyranoside (IPTG; 0.7 mM; Gold Biotechnology) and δ-aminolevulinic acid (1 mM; Acros Organics) were added and then the *E. coli* culture was incubated at 25 °C and 200 rpm for 48 h. The cells were harvested by centrifugation at 6675 g for 30 min. Protein purification was performed as described previously [28]. The molecular weight and purity of the proteins were confirmed by SDS-PAGE.

The sequence encoding the C-terminal His-tagged *Trypanosoma brucei* NADPH-cytochrome P450 reductase (TbCPR; XP_828912.1) was incorporated into the pCWori vector and confirmed by Sanger sequencing. The protein expression procedure was similar to the one for CYP enzymes and the only difference was that IPTG (0.7 mM) and riboflavin (4 μg/mL) were added when OD_600_ was 0.6. The purification procedure was also modified from the one for CYP enzymes. TbCPR was purified by Ni-NTA chromatography followed by dialysis for 24 h in the buffer containing 50 mM Tris (pH 7.6), 10% glycerol, 0.1 mM EDTA, and 1 mM DTT. The molecular weight and purity of TbCPR were confirmed by SDS-PAGE.

### Western blot analysis

Polyclonal antisera of CYP5122A1 and CYP51 were raised against the purified recombinant proteins in rats (a gift from Dr. Jianming Qiu of the University of Kansas Medical Center). To determine the levels of CYP5122A1, CYP51, lipophosphoglycan (LPG), and proteophosphoglycan (PPG), promastigote lysates were boiled in SDS-containing sample buffer at 95–100 °C for 5 min and resolved by SDS-PAGE. After transfer to a PVDF membrane, blots were probed with rat anti-CYP5122A1 (1:500), rat anti-CYP51 (1:500), or monoclonal antibody CA7AE (1:1000, for LPG and PPG) [29] followed by HRP-conjugated anti-rat or anti-mouse secondary antibodies. Antibody to alpha-tubulin (Thermo Fisher Scientific) was used as the loading control. Similar experiments were performed to determine the LPG and PPG levels in the culture supernatant of stationary phase promastigotes.

### UV-Vis spectroscopy

The ferric absolute spectra, carbon monoxide (CO) difference spectra, and substrate binding difference spectra for CYP51 and CYP5122A1 were recorded on a Cary 3500 UV/Vis spectrophotometer (Agilent) by following published protocols [30]. The active P450 content and the binding constant of sterol substrates were determined as described by Hargrove et al. [22].

### Reconstitution of CYP51 and CYP5122A1 catalytic activity

The standard reconstitution reaction (100 μL) contained 1 μM CYP enzyme, 5 μM TbCPR, 50 μM substrate, and 50 μg/mL phospholipids (DLPC:DMPC:DMPG = 5:4:1, w/w/w) in 100 mM phosphate buffer (pH 6.2) with 3.3 mM MgCl_2_. The reaction was initiated with the addition of 1 mM NADPH and then incubated at 37 °C for up to 1 h. To obtain the metabolites of lanosterol in the CYP5122A1-catalyzed reaction for NMR analysis, the reaction mixture was slightly modified, which contained the 3 μM CYP enzyme, 12 μM TbCPR, 30 μM lanosterol, and 50 μg/mL phospholipids in the same buffer (4 mL each reaction and a total of 385 mL). After adding 1 mM NADPH, the reaction was incubated at 37 °C for 2 h. The sterols in the reconstitution reactions were extracted and analyzed by liquid chromatography-tandem mass spectrometry (LC-MS/MS) as previously described [16].

### Sterol purification

The crude sterol sample was dried under a vacuum using a rotary evaporator and redissolved in 1:1 (v/v) DMSO: tetrahydrofuran. Sterol purification was performed using an Agilent 1260 Infinity II HPLC system with a G7157A Agilent prep autosampler, a G7161A Agilent binary pump, a G7115A Agilent diode array detector, and a G1364E Agilent fraction collector. The sterols were separated on a Waters XBridge BEH C18 OBD prep column (19 mm × 250 mm, 5 μm). LC mobile phases consisted of (A) water containing 0.05% (v/v) difluoroacetic acid and (B) acetonitrile containing 0.05% (v/v) difluoroacetic acid. A gradient elution was used for purification, which began from 75% to 100% B over 10 min and held at 100% B for 35 min with a flow rate of 20 mL/min. The detection wavelength was set at 214 nm and 254 nm.

### NMR analysis

All NMR spectra were acquired on an Avance AVIII 500 MHz spectrometer equipped with a multinuclear BBFO cryoprobe. Approximately 2 mg of the purified sterol metabolite was dissolved in 0.5 mL of deuterated chloroform (CDCl_3_). NMR experiments including ^1^H, ^13^C, DEPT, COSY, HSQC, and HMBC were recorded with extended runtimes. Chemical shifts are reported in ppm, and coupling constants are reported in Hz. The chemical shifts at 7.26 ppm and 77.15 ppm for proton and carbon spectra respectively are from residual CHCl_3_ in CDCl_3_ and were used as internal references. ^1^H and ^13^C NMR assignments of lanosterol were used as a reference for the structural assignment of the proposed sterol metabolite.

### CYP inhibition assay

The inhibition assays for CYP5122A1 and CYP51 were carried out as described previously for leishmanial CYP51 [28]. All tested compounds were dissolved in DMSO except miltefosine, pentamidine isethionate, paromomycin sulfate, and amphotericin B deoxycholate which were dissolved in water. Briefly, a CYP enzyme (50 nM) was incubated with the fluorogenic substrate 7-benzyloxy-4-trifluoromethylcourmarin (BFC; 50 μM) and various concentrations of test compounds in 100 mM phosphate buffer (pH 7.4) and 3.3 mM MgCl_2_. Compound solvents (DMSO and water) were used as the negative control. The reaction was initiated with the addition of cumene hydroperoxide (100 μM), incubated at 37 °C, and monitored at an emission wavelength of 538 nm and an excitation wavelength of 410 nm on a Tecan Infinite^®^ M200 Pro microplate reader. For test compounds, the percentage of inhibition at each concentration was calculated as $(1-{RFU}_{\text{compound}}/{RFU}_{\text{control}})\times 100$, where RFU is the relative fluorescence unit. A plot of the percentage of inhibition versus the logarithm of the compound concentration was fit with the following two-parameter logistic equation to obtain the IC_50_ value (GraphPad Prism version 8.4.0):

$$y=\frac{100}{1+{\left(\frac{x}{{IC}_{50}}\right)}^{\text{Hill}\;\text{Slope}}}$$

### *Leishmania donovani* 1S2D culture and genetic manipulations of *CYP5122A1*

*Leishmania donovani* strain 1S2D (MHOM/SD/62/1S-CL2D) clone LdBob promastigotes were cultivated at 27 °C in a complete M199 medium (M199 with 10% heat-inactivated fetal bovine serum and other supplements, pH 7.4) [31]. To monitor growth, culture densities were measured daily using a Beckman Z2 Cell Counter. Log phase promastigotes refer to replicative parasites at densities <1.0 × 10^7^ cells/ml, and stationary phase promastigotes refer to non-replicative parasites at densities >2.0 × 10^7^ cells/ml.

To delete chromosomal *CYP5122A1* alleles, the upstream and downstream flanking sequences of *CYP5122A1* (~1 Kb each) were amplified by PCR and cloned in the pUC18 vector. Genes conferring resistance to blasticidin (*BSD*) and puromycin (*PAC*) were cloned between the upstream and downstream flanking sequences to generate pUC18-KO-*CYP5122A1*:BSD and pUC18-KO-*CYP5122A1*:PAC, respectively. To generate the *CYP5122A1*+/− heterozygotes (*ΔCYP5122A1::BSD/CYP5122A1*), wild-type (WT) *L. donovani* promastigotes were transfected with linearized *BSD* knockout fragment (derived from pUC18-KO-*CYP5122A1*:BSD) by electroporation and transfectants showing resistance to blasticidin were selected and later confirmed to be *CYP5122A1*+/− by Southern blot as previously described [32]. To delete the second chromosomal allele of *CYP5122A1*, we used an episome-assisted approach as previously described for other genes [33]. First, the *CYP5122A1* open reading frame (ORF) was cloned into the pXNG4 vector to generate pXNG4-*22A1* and introduced into *CYP5122A1*+/− parasites. The resulting *CYP5122A1*+/− +pXNG4-*22A1* cell lines were then transfected with linearized *PAC* knockout fragment (derived from pUC18-KO-*CYP5122A1*:PAC) and selected with 15 μg/ml of blasticidin, 15 μg/ml of puromycin and 150 μg/ml of nourseothricin. The resulting *CYP5122A1* chromosomal null mutants with pXNG4-*522A1* (*ΔCYP5122A1::BSD/ΔCYP5122A1::PAC* + pXNG4-*CYP5122A1* or *Cyp5122A1*^−^+ pXNG4-*22A1*) were validated by Southern blot as previously described for other genes [32].

### Determination of in vitro antileishmanial activities of antifungal azole compounds in *CYP5122A1* mutants of *L. donovani* strain 1S2D

Stock solutions for test compounds were prepared at 10 mM in DMSO. To measure the antileishmanial activity of these inhibitors, log phase promastigotes were inoculated in complete M199 media at 2.0 × 10^5^ cells/ml in 24-well plates (1 ml/well). Inhibitors were added to various concentrations and control wells contained DMSO only (0.1–0.5%). Culture densities were determined after 48 h using a Beckman Z2 Cell Counter.

### Promastigote essentiality assay

*L. donovani* WT, *CYP5122A1*+/− +pXNG4-*22A1*, and *Cyp5122A1*^−^ + pXNG4-*22A1* promastigotes were inoculated in complete M199 media at 1.0 × 10^5^ cells/ml in the presence or absence of 50 μg/ml of GCV (the negative selection agent) or 150 μg/ml of nourseothricin (the positive selection agent). Every three days, cells were reinoculated into fresh media with the same negative or positive selection agents, and percentages of GFP-high cells for each passage were determined by flow cytometry using an Attune NxT Acoustic Flow Cytometer. After 14 passages, individual GFP-high and GFP-low clones of *Cyp5122A1*^−^ + pXNG4-*22A1* were isolated by fluorescence-activated cell sorting (FACS), followed by serial dilution in 96-well plates and expanded in the presence of GCV and absence of nourseothricin. via serial dilution in 96-well plates. The GFP levels of selected clones were determined by flow cytometry.

### Mouse infection

Female BALB/c mice (7–8 weeks old) were purchased from Charles River Laboratories International (Wilmington, MA). All animal procedures were performed as per approved protocol by Animal Care and Use Committee at Texas Tech University (PHS Approved Animal Welfare Assurance No A3629-01). To determine whether CYP5122A1 is required during the intracellular amastigote stage, day 3 stationary phase promastigotes were injected into the peritoneal cavity of BALB/c mice (5.0 × 10^8^ cells/mouse, 10 mice per group). For each group, starting from day one post infection, one-half of the mice received GCV at 7.5 mg/kg/day for 14 consecutive days (0.5 ml each, intraperitoneal injection), while the other half (control group) received an equivalent volume of sterile PBS. At 4- or 7-weeks post infection, mice were euthanized through a controlled flow of CO_2_ asphyxiation and infected spleens were isolated and homogenized. Parasite numbers in spleen homogenates were determined by limiting dilution assay [34] or qPCR as described below.

### Quantitative PCR (qPCR)

To determine parasite loads in infected mice, genomic DNA was extracted from spleen homogenate and qPCR reactions were run in triplicates using primers targeting the 28S rRNA gene of *L. donovani* [32]. Cycle threshold (Ct) values were determined from melt curve analysis. A standard curve of Ct values was generated using serially diluted genomic DNA samples from *L. donovani* promastigotes (from 0.1 cell/reaction to 10^5^ cells/reaction) and Ct values >30 were considered negative. Parasite numbers in spleen samples were calculated based on their Ct values using the standard curve. Control reactions included sterile water and DNA extracted from the uninfected mouse spleen.

To determine pXNG4-*22A1* plasmid levels in promastigotes and amastigotes, a similar standard curve was generated using serially diluting pXNG4-*22A1* plasmid DNA (from 0.1 copy/reaction to 10^5^ copies/reaction) and primers targeting the *GFP* region. qPCR was performed with the same set of primers on DNA samples from promastigotes or spleen and the average plasmid copy number per cell was determined by dividing the total plasmid copy number by the total parasite number based on Ct values.

To determine the transcript levels of *SHERP* (small hydrophilic endoplasmic reticulum-associated protein), total RNA was extracted from promastigotes and converted into cDNA using a high-capacity reverse transcription kit (Bio-Rad), followed by qPCR using primers targeting *SHERP* or 28S rRNA genes. The relative expression level of *SHERP* was normalized to that of 28s rRNA using the 2^−ΔΔ(Ct)^ method [35]. Control reactions were carried out without leishmanial RNA and without reverse transcriptase.

### *Leishmania donovani* 1S2D stress response assays

*L. donovani* promastigotes were cultivated in complete M199 media (pH 7.4) at 27 °C until they reach the stationary phase. For heat tolerance, promastigotes were incubated at 37 °C. To test their sensitivity to acidic pH, promastigotes were transferred to a pH 5.0 medium (same as the complete M199 medium except that the pH was adjusted to 5.0 using hydrochloric acid). For starvation response, promastigotes were transferred to PBS (pH 7.4). For resistance to oxidative or nitrosative stress, parasites were incubated in various concentrations of H_2_O_2_ or S-nitroso-N-acetylpenicillamine (SNAP). Cell viability was determined at the indicated times by flow cytometry after staining with 5 μg/ml of propidium iodide. Parasite growth was monitored using a Beckman Z2 Cell Counter.

### Growth inhibition and sterol analysis experiments with *L. donovani* LV82 parasites

*L. donovani* LV82 (MHOM/ET/67/LV82) promastigotes were assayed to examine the effects of azole drugs on parasite growth and sterol composition according to methods detailed by Feng et al. [16]. In brief, low-pass LV82 promastigotes obtained by transforming amastigotes from infected hamster spleens were cultured in Schneider’s *Drosophila* medium (Gibco) containing 25% heat-inactivated fetal bovine serum (Sigma-Aldrich) and penicillin-streptomycin (Gibco). Growth assays were performed after three-day incubation of promastigotes at 26 °C with or without azole drugs in the above medium containing 50 U/mL penicillin and 50 μg/mL streptomycin using 3-(4,5-dimethylthiazol-2-yl)-5-(3-carboxymethoxyphenyl)-2-(4-sulfophenyl)-2H-tetrazolium, inner salt (MTS):phenazine methosulfate (PMS) as an indicator of parasite growth as described earlier [16]. Absorbance values for individual wells measured at 490 nm were normalized to positive and negative controls, then normalized absorbance values were plotted against concentration. Normalized data were fitted to the equation y=m1+(m2-m1)/(1+(x/m3)^m4^) in KaleidaGraph v4.5 (Synergy Software); absolute EC_50_ values were then calculated as described by Joice et al [36] with standard deviations calculated using R (drc) package [37]. At least three independent experiments were used to determine the effect of different azole concentrations on parasite growth. Validity criteria for experiments were as outlined in Feng et al. [16]. Samples for sterol analysis were obtained after incubating LV82 promastigotes treated for 24 hr at 26 °C with either vehicle (0.4% DMSO v/v), clotrimazole (20 μM), or voriconazole (50 μM). Promastigotes exposed to clotrimazole were maintained in Schneider’s *Drosophila* medium containing 25% heat-inactivated FBS containing 100 U/mL penicillin and 100 μg/mL streptomycin and promastigotes exposed to voriconazole were cultured in Schneider’s *Drosophila* medium containing 25% heat-inactivated FBS containing 50 U/mL penicillin and 50 μg/mL streptomycin. After exposure to azole drugs or vehicle, parasites were centrifuged, washed with PBS, and stored at −80 °C prior to analysis as mentioned earlier [16].

## Results

### Purified CYP51 and CYP5122A1 exhibited characteristic spectral properties of a CYP enzyme.

**Figure 1.**
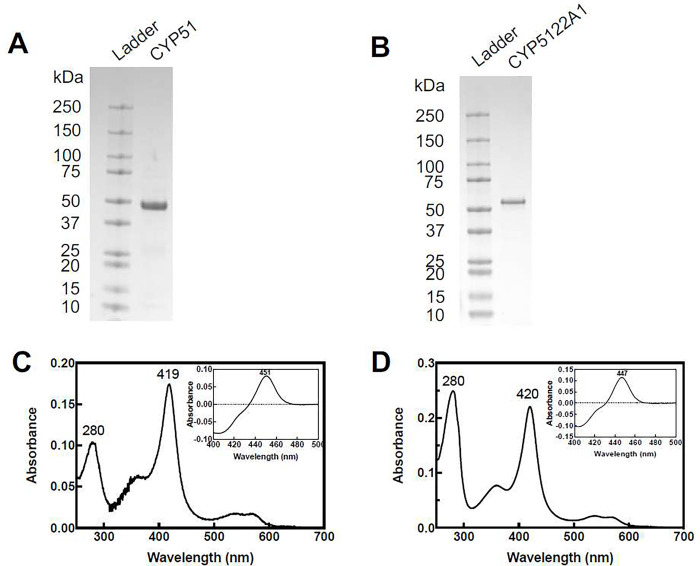
Purified CYP51 and CYP5122A1 exhibited characteristic spectral properties. (A-B) SDS-PAGE analysis of recombinant CYP51 and CYP5122A1. The absolute absorbance spectra were measured for CYP51 (C) and CYP5122A1 (D), showing the Soret band at around 420 nm. The insets show the CO difference spectra of sodium dithionite-reduced CYPs which exhibited the characteristic peak at around 450 nm.

*L. donovani* CYP5122A1 and CYP51 have an N-terminal transmembrane domain anchored to the endoplasmic reticulum, which needs to be removed from the recombinant protein constructs to facilitate expression and purification. We tested several truncations of CYP5122A1, and the removal of the first 60 amino acids from the N-terminus yielded the best expression results (data not shown). Recombinant CYP51 (removal of the first 31 amino acids from the N-terminus) was designed based on a previous study on the N-terminal truncated *L. infantum* CYP51 [22]. After expression and chromatographic purification, recombinant CYP5122A1 and CYP51 were obtained at high purity as indicated by the SDS-PAGE result (Fig. 1A–B). Subsequent Western blot analysis of CYP5122A1 (Supplementary Fig. 2) showed that the recombinant protein had slightly lower molecular weight compared with the wild-type proteins detected in *Leishmania* spp. because of the deletion of the transmembrane domain.

CYP enzymes have an iron-containing heme as a prosthetic group whose different oxidation states can be characterized using UV-Vis spectroscopy. Results of spectral analysis demonstrated that the two proteins possessed properties typical of a CYP enzyme. Purified CYP5122A1 and CYP51 were in the oxidized state and displayed the Soret peak at 420 nm and 419 nm, respectively (Fig. 1C–D). Upon reduction with dithionite and binding to CO, both enzymes exhibited the Soret peak at around 450 nm and almost no absorption at 420 nm in the presence of lanosterol (insets of Fig. 1C–D). This indicated that they existed in the catalytically active P450 form rather than the inactive P420 form.

In the natural context, a CYP enzyme requires two electrons during a catalytic cycle. The electrons are donated by the cofactor NADPH and transferred to the CYP sequentially by its redox partner, CPR. However, three putative CPRs (XP_003862234.1, XP_003864839.1, and XP_003864409.1; protein sequence identity ranges from 21% to 27% between them) have been reported in the *L. donovani* genome and none has been studied or characterized to our knowledge. Previously, a recombinant CPR from another trypanosomatid *T. brucei* (TbCPR) has been characterized for its P450 reduction activity and used as a surrogate CPR to reconstitute catalytic activities of CYP enzymes from *Leishmania* and *T. cruzi* [22, 38, 39]. Here, in the presence of NADPH, TbCPR was able to transfer the first electron to leishmanial CYP5122A1 and CYP51, and the resulting reduced CO difference spectra (Supplementary Fig. 3) were similar to the ones reduced by sodium dithionite. Therefore, TbCPR may be used as a surrogate redox partner in place of the endogenous leishmanial CPR for studying the catalytic activities of the two leishmanial CYP enzymes, although exact biochemical roles of the three putative leishmanial CPRs warrant future investigations.

### CYP5122A1 had binding specificity to C4-methylated sterols.

**Figure 2.**
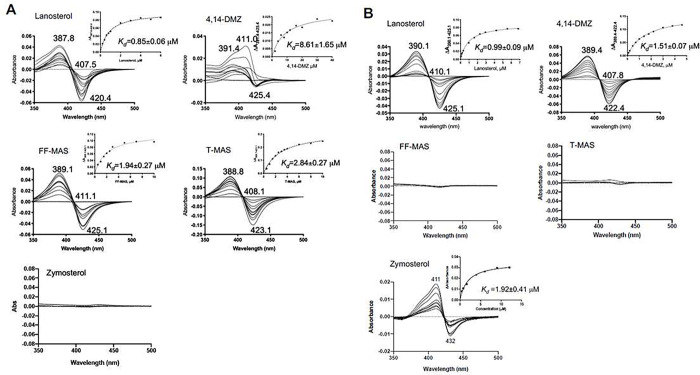
Binding spectra of sterols with CYP5122A1 (A) and CYP51 (B). Purified recombinant CYPs (in the oxidized ferric form) were incubated with an increasing amount of sterol ligands and difference spectra were recorded over a reference sample that only contained protein and buffer. Dissociation constant (K_d_) was derived by fitting the equation ΔA = ΔA_max_[L]/(K_d_ + [L]) to the peak-to-trough absorbance difference (ΔA) versus the ligand concentration ([L]) curve, where ΔA_max_ is the maximal amplitude of the spectral response.

When a substrate binds to the CYP enzyme, it displaces the distal H_2_O ligand of the heme iron, converting the heme iron from the low-spin state to the high-spin state. Such binding mode is termed “type I binding” and will yield a UV-Vis difference spectrum with a peak at 390 nm and a trough at 420 nm [30]. This unique feature was employed for identifying potential substrates of CYP5122A1. Five intermediate sterols within the ergosterol biosynthetic pathway (lanosterol, 4,14-DMZ, FF-MAS, T-MAS, and zymosterol; Scheme 1) were tested due to their same tetracyclic ring structure, only differing in the number of methyl groups at C4 and/or C14 positions. Among them, sterols with at least one C4-methyl group (lanosterol, 4,14-DMZ, FF-MAS, and T-MAS) showed type I binding to CYP5122A1 (Fig. 2A), regardless of whether the C14-methyl group is present (lanosterol and 4,14-DMZ) or absent (FF-MAS and T-MAS). Zymosterol, which has no C4- or C14-methyl group, did not induce any appreciable change in the difference binding spectrum (Fig. 2A). These results suggest that C4-methylated sterols like lanosterol, 4,14-DMZ, FF-MAS, and T-MAS may serve as substrates of CYP5122A1. In comparison, CYP51, which is known to act as a C14-demethylase, exhibited a binding specificity distinct from CYP5122A1 (Fig. 2B). Only sterols with a C14-methyl group (lanosterol and 4,14-DMZ) showed typical type I binding to CYP51, consistent with a previous report [22]. The C14-demethylated sterols (FF-MAS and T-MAS) did not elicit any change in their difference binding spectra. Interestingly, zymosterol, which lacks both C4- and C14-methyl groups, produced an atypical difference binding spectrum with a peak at 411 nm and a trough at 432 nm (Fig. 2B), which differs from the typical binding spectral changes, i.e., type I, II and reverse type I (also called pseudo or modified type II) [40]. Similar atypical spectral changes were also observed for the binding of racemic and *S*-bicalutamide to CYP46A1 through the bridging water ligand [41]. This suggests that zymosterol can enter into the binding pocket of CYP51, albeit unable to dislodge the hexacoordinated water molecule.

### CYP5122A1 acted to oxidize the C4-methyl group of sterols in *Leishmania*.

**Figure 3.**
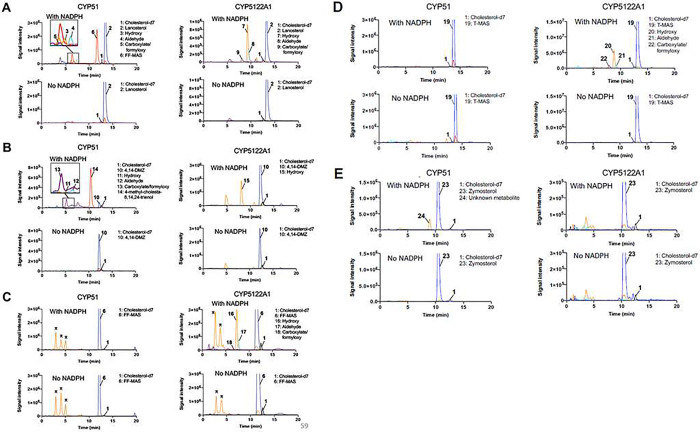
LC-MS/MS chromatograms of CYP reconstitution assays using (A) lanosterol, (B) 4,14-DMZ, (C) FF-MAS, (D) T-MAS, and (E) zymosterol as the sterol substrates. The peaks of the internal standard cholesterol-d7 (black), substrates (blue), hydroxy metabolites (orange), aldehyde metabolites (cyan), carboxylate/formyloxy metabolites (purple), and 14-demethylated products (red) were indicated in the chromatograms. Peaks with asterisk were detected in both incubations with or without NADPH and hence considered as background signals.

Using TbCPR as the redox partner, the catalytic activities of CYP5122A1 and CYP51 were reconstituted *in vitro* with the addition of NADPH. Interestingly, the optimal reaction pH was pH 6.2–6.6 for both enzymes, rather than physiological pH (Supplementary Fig. 4). Metabolite profiles were analyzed by comparing LC-MS/MS chromatograms of the reactions in the presence and absence of NADPH. As expected, CYP51 catalyzed the C14-demethylation of lanosterol and 4,14-DMZ and converted them to FF-MAS and 4-methylzymosterol, respectively (Fig. 3A and 3B). The alcohol, aldehyde, and carboxylate/formyloxy [42] intermediate metabolites generated during the sequential oxidation were also detected. The LC-MS/MS MRM (multiple reaction monitoring) transitions used for monitoring the intermediate oxidation metabolites of lanosterol and 4,14-DMZ were: 425>109 and 411>109 (hydroxy), 423>109 and 409>109 (aldehyde), 439>109 and 397>95 (carboxylate/formyloxy), respectively, based on the dehydrated molecular ions and common fragment ions of sterols [16]. In contrast, no oxidation metabolites were detected in the reactions of FF-MAS and T-MAS with CYP51 (Fig. 3C and 3D). The MRM transitions used for monitoring the intermediate oxidation metabolites of FF-MAS and T-MAS were: 409>109 and 411>109 (hydroxy), 425>109 and 409>109 (aldehyde), 423>109 and 425>109 (carboxylate/formyloxy), respectively. In addition, zymosterol was oxidized by CYP51 to form a hydroxylated metabolite, which was detected in the MRM transition of 383>95 (Fig. 3E). This is consistent with the atypical difference binding spectrum of zymosterol and CYP51 observed above, although the exact location of hydroxylation remains unknown, presumably on or near C14.

When compared to CYP51, CYP5122A1 clearly showed a different substrate specificity and formed oxidation metabolites of different identities. Sterols that exhibited type I binding spectral change with CYP5122A1 (i.e., lanosterol, 4,14-DMZ, FF-MAS, and T-MAS) were also metabolized by this enzyme, yielding a mixture of alcohol, aldehyde, and/or carboxylate/formyloxy metabolites of the corresponding sterol substrate (Fig. 3A–D), whereas none of the zymosterol oxidation metabolites was detected (Fig. 3E). Importantly, the oxidation metabolites of lanosterol formed by CYP5122A1 had different LC retention times from those formed by CYP51. For example, the alcohol metabolite formed by CYP5122A1 (peak **7** in Fig. 3A) was eluted at 9.6 min, whereas the alcohol metabolite formed by CYP51 (peak **3** in Fig. 3A) was eluted at 7.1 min. This indicates that CYP5122A1 did not catalyze the same C14 methyl oxidation as CYP51 did. In contrast to CYP51, CYP5122A1 converted FF-MAS and T-MAS (both are C4-dimethylated and C14-demethylated sterols) to their corresponding alcohol, aldehyde, and carboxylate/formyloxy metabolites (Fig. 3C and 3D). Additionally, it was observed that CYP5122A1 converted 4,14-DMZ (a C4a-monomethylated and C14-methylated sterol) to an alcohol metabolite, but no aldehyde or carboxylate/formyloxy metabolite was detected (Fig. 3B). This result, along with the weaker binding of 4,14-DMZ to CYP5122A1 (Fig. 2A), suggests that CYP5122A1 may not be the optimal enzyme to catalyze the second C4-demethylation reaction and other enzymes like leishmanial Erg25 may still play a role, which warrants future investigations. No oxidation metabolite formation was seen for zymosterol with CYP5122A1. As expected, NADPH was required for the catalytic activity of both CYP51 and CYP5122A1 (Fig. 3). Taken together, these findings prompted us to propose CYP5122A1 as a sterol C4-methyl oxidase or a sterol C4-demethylase, assuming that the carboxylate metabolites formed by CYP5122A1 will be decarboxylated and reduced by a dehydrogenase/decarboxylase and a 3-ketosterol reductase, respectively.

**Figure 4.**
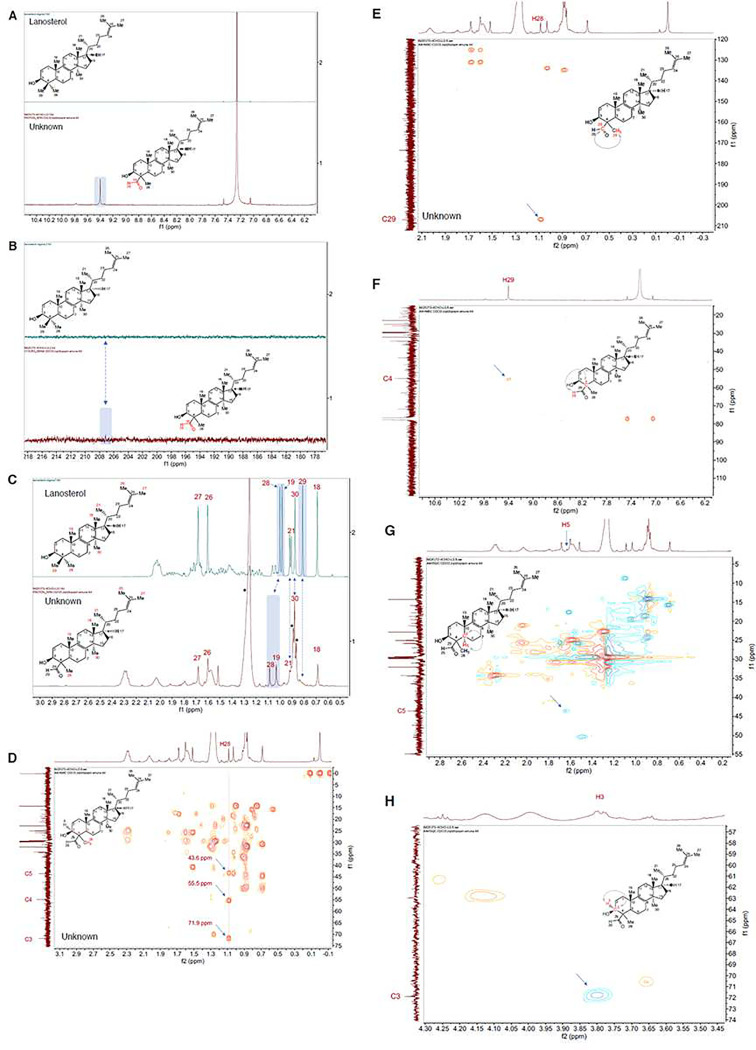
The 1D and 2D NMR spectra of the purified sterol metabolite. (A-B) ^1^H and ^13^C NMR spectral comparison between lanosterol (reference) and the purified sterol metabolite indicating the presence of the aldehyde group in the sterol metabolite. (C) ^1^H NMR comparison of chemical shifts for methyl groups between lanosterol (reference) and the purified sterol intermediate. (D-E) HMBC correlation for 28-Me. (F) HMBC correlation between C4 and aldehyde proton H29. (G) HSQC correlations for C5. (H) HSQC correlations for C3.

To fully elucidate the biochemical role of CYP5122A1, its reaction with lanosterol was scaled up from 0.1 mL to 385 mL so that sufficient amounts of oxidation metabolites could be isolated, purified, and analyzed by NMR spectroscopy for structural identification. The 1D and 2D NMR spectra of the aldehyde metabolite (2 mg; referred to as “unknown”) were successfully collected. The ^1^H and ^13^C NMR assignments of lanosterol were used as a reference for the structural assignment of the unknown lanosterol metabolite. The singlet at 9.4 ppm in the ^1^H spectrum and the signal appearing at 207 ppm in the ^13^C spectrum indicate the presence of the aldehyde group in the molecule (Fig. 4A–B). The hydrogen atoms on the methyl groups 18 (0.68 ppm, s), 21 (0.91 ppm, d), 26 (1.60 ppm, s), and 27 (1.68 ppm, s) in the unknown compound exhibited chemical shifts identical to those in lanosterol (Fig. 4C). However, the hydrogen atoms on 19-Me (1.03 ppm, s) and 28-Me (1.09 ppm, s) had a downfield shift, indicating the presence of a nearby electron-withdrawing group. Noteworthy, the ^1^H signal associated to 29-Me of lanosterol (0.81 ppm, s, lanosterol) was not observed in the ^1^H spectrum of the unknown compound as shown by the dotted arrow in Fig. 4C. Moreover, the signal associated with 30-Me (0.88 ppm, s) is present in both ^1^H spectra of lanosterol and the unknown compound (Fig. 4C). These results alluded to a structure containing an aldehyde group that replaces one of the methyl groups at the C-4 position. The structural assignment was confirmed by the HMBC spectrum which displayed the correlations from H-28 to C-3, C-4, C-5, and C-29 (Fig. 4D–E) and from H-29 to C-4 (Fig. 4F). Unfortunately, the stereochemistry of the unknown compound at the C-4 position could not be ascertained due to the low signal intensities in the NOESY spectrum (data not shown). In addition to C-4, the assignment of C-3 and C-5 was confirmed by the HSQC correlations between C-3 and C-5 with their corresponding protons H-3 and H-5 (Fig. 4G–H). However, it should be noted that not all of the observed signals in the NMR spectra were contributed by the aldehyde metabolite. In the ^1^H NMR spectrum (Fig. 4C), the broad peak at 1.26 ppm and the multiplet at 0.86 ppm (indicated by asterisks) were caused by the contamination of grease. The broad peak at 1.56 ppm corresponded to water. The singlet at 1.51 ppm was due to an unknown impurity present in the sample and was not related to the molecule of interest. Taken together, these NMR spectroscopy and LC-MS/MS results clearly indicate that CYP5122A1 catalyzes the sequential oxidation reaction of the lanosterol C4 methyl groups, leading to C4-demethylation during ergosterol biosynthesis in *Leishmania*.

### CYP5122A1 was essential for *L. donovani* promastigotes in culture.

To assess the essentiality of *CYP5122A1*, we applied a complementing episome-assisted knockout approach coupled with negative selection (Supplementary Fig. 5A) [43]. Briefly, we first replaced one allele of the *CYP5122A1* gene with the *BSD*-resistance cassette to generate the *Ld22A1*+/− mutant by homologous recombination. The heterozygotes (half knockout) were then episomally transfected with pXNG4-*22A1* carrying *SAT*, *GFP*, and *TK*, followed by the replacement of the second allele of *CYP5122A1* with the *PAC*-resistance cassette to generate the null mutant *Ld22A1*^−^ +pXNG4-*22A1* (Supplementary Fig. 5B-C). Promastigotes were able to proliferate in complete M199 medium after these genetic manipulations, though *Ld22A1*+/− +pXNG4-*22A1* and *Ld22A1*^−^ +pXNG4-*22A1* exhibited delayed growth *in vitro* (Supplementary Fig. 6A). They attained the same plateau densities as Ld1S WT and *Ld22A1*+/− on day 5 (one day later). The obtained mutants were assessed at the protein level by Western blot analysis (Supplementary Fig. 6B-C). Compared to Ld1S WT, *Ld22A1*+/− showed a reduced level of CYP5122A1 (*p* < 0.01), whereas the heterozygous and null mutants transfected with pXNG4-*22A1* had overexpression of CYP5122A1 (*p* < 0.001). Meanwhile, the genetic manipulation of *CYP5122A1* did not affect the CYP51 expression significantly (Supplementary Fig. 6B-C).

**Figure 5.**
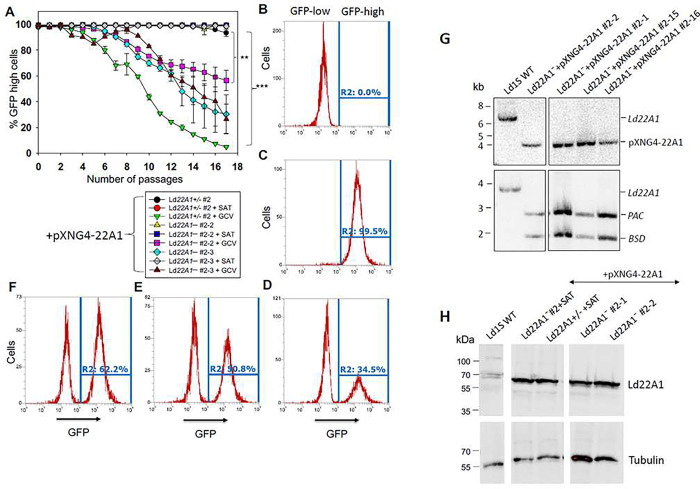
CYP5122A1 is indispensable during the promastigote stage. (A) Promastigotes were continuously cultivated in the presence or absence of ganciclovir (GCV) or nourseothricin (SAT) and passed every three days. Percentages of GFP-high cells were determined for every passage. Error bars represent standard deviations from three repeats (**: *p* < 0.01, ***: *p* < 0.001). (B-D) After 14 passages, WT (B) and *Ld22A1*^−^+pXNG4-22A1 #2 parasites grown in the presence of SAT (C) or GCV (D) were analyzed by flow cytometry to determine the percentages of GFP-high cells (indicated by R2 in the histograms). (E-F) Two clones (E: #2-1; F: #2-2) were isolated from the GFP-low population in D by FACS followed by serial dilution and amplification in the presence of GCV and analyzed for GFP expression by flow cytometry. (G) To verify the presence of episomal *Ld22A1* allele, single clones isolated from the GFP-low (#2-1 and #2-2) and GFP-high (#2-15 and #2-16) populations of *Ld22A1*^−^+pXNG4-22A1 #2 parasites (D) were expanded in the presence of GCV and examined by Southern blot using the *Ld22A1* ORF probe (top) or an upstream flanking region probe (bottom). Bands corresponding to endogenous *Ld22A1*, episomal *Ld22A1*, and antibiotic resistance markers *PAC/BSD* were indicated. (H) To examine the CYP5122A1 protein levels, whole cell lysates from log phase promastigotes before and after sorting were analyzed by western blot using anti-LdCYP5122A1 (top) or anti-a-tubulin antibodies.

To determine if CYP5122A1 is required for promastigotes, *Ld22A1*+/− +pXNG4-*22A1* and *Ld22A1*^−^ +pXNG4-*22A1* (clone #1 and #2) were cultivated in the absence or presence of selective drugs (nourseothricin as the positive selection drug to retain pXNG4-*22A1* and GCV as the negative selection drug to expel plasmid) and analyzed for GFP expression in each passage as a readout for plasmid retention (Fig. 5). When growing in media containing nourseothricin (+SAT), all cell lines maintained high levels of GFP fluorescence (> 95% GFP-high) through multiple passages as expected (Fig. 5A–C). Without nourseothricin or GCV, the heterozygous mutant and clone #1 of the chromosomal-null mutant retained high levels of GFP, whereas the percentage of GFP-high population in clone #2 of the chromosomal-null mutant gradually decreased to ~30% after 17 passages. These findings allude to clonal variations among the CYP5122A1 mutants in their ability to retain pXNG4-*22A1* in the absence of selective pressure. When cultivated in the presence of GCV and absence of nourseothricin, cells would favor eliminating the plasmid to avoid toxicity if the plasmid did not contain any essential genes. Conversely, if there was an essential gene on the plasmid, cells would retain the plasmid despite the associated cost [43]. As shown in Fig. 5A, *Ld22A1*+/− +pXNG4-*22A1* promastigotes progressively lost their GFP expression to < 5% GFP-high by passage 17 in GCV, indicating that pXNG4-*22A1* was dispensable in this cell line which had one *CYP5122A1* endogenous allele. In comparison, GCV treatment lowered the percentage of GFP-high population in *Ld22A1*^−^ +pXNG4-*22A1* to a much lesser extent (~58% and ~30% for clone #1 and #2, respectively; Fig. 5A and D). To examine if the plasmid was required for the survival and proliferation of *Ld22A1*^−^ +pXNG4-*22A1*, we separated GFP-high and GFP-low cells from *Ld22A1*^−^ +pXNG4-*22A1* clone #2 grown in the presence of GCV (Fig. 5D) by FACS and then isolated two single clones of each population via serial dilution. Importantly, clones isolated from the GFP-low population (#2-1 and #2-2) quickly regained GFP expression after two rounds of amplification (+GCV, −SAT) (Fig. 5E–F). Southern blot analysis verified that clones from both GFP-high (#2-15 and #2-16) and GFP-low (#2-1 and #2-2) populations had retained the pXNG4-*22A1* plasmid (Fig. 5G). Finally, western blots revealed robust expression of CYP5122A1 in these sorted clones at levels similar to *Ld22A1*+/− +pXNG4-*22A1* and *Ld22A1*^−^ +pXNG4-22A1 grown in the presence of SAT. Together, these results supported that CYP5122A1 is essential for *L. donovani* in the promastigote stage.

### CYP5122A1 was essential for *L. donovani* amastigotes in mice.

**Figure 6.**
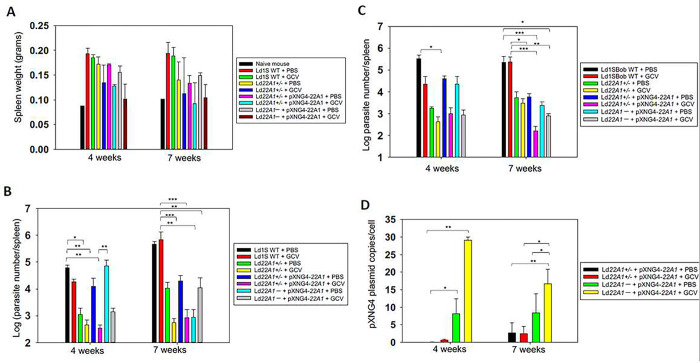
CYP5122A1 is indispensable during the amastigote stage. BALB/c mice were infected (i.p.) with day 3 stationary phase promastigotes (5 × 10^8^ cells/mouse) and treated with GCV or PBS as described in Materials and Methods. (A) Spleen weights from uninfected and infected mice were measured at 4- or 7-weeks post infection. (B-C) Parasite numbers in infected spleens were determined by qPCR (B) or limiting dilution assay (C) at the indicated times. (D) The pXNG4-22A1 plasmid copy numbers in amastigotes were determined by qPCR (#/cell ± SDs). Error bars represent standard deviations from three repeats (*: *p* < 0.05, **: *p* < 0.01, ***: *p* < 0.001).

To investigate whether CYP5122A1 is required during the amastigote stage, we infected the BALB/c mice with day 3 stationary phase promastigotes and treated them with either PBS (solvent control) or GCV. The weights of spleens from uninfected and infected mice were measured at 4- or 7-weeks post infection as a readout for splenomegaly, a symptom of VL (Fig. 6A). Parasite loads in infected spleens was determined by qPCR analysis (Fig. 6B). As expected, the infection with Ld1S WT increased spleen weight in mice and the parasite growth was not affected by GCV treatment at weeks 4 and 7 post infection. Compared with the WT-infected group, the parasite loads in mice infected by *Ld22A1*+/− were significantly lower (*p* < 0.05), indicative of attenuated virulence. This could be partially reversed by the episomal expression of *CYP5122A1*, as we detected in mice infected by *Ld22A1*+/− +pXNG4-*22A1* or *Ld22A1*^−^ +pXNG4-*22A1* with PBS treatment at week 4 post infection (Fig. 6A–B). GCV treatment reduced the splenomegaly and parasite loads at week 4 post infection, but not week 7 post infection for *Ld22A1*^*−*^ +pXNG4-*22A1*. It is possible that the effect of GCV (administered during the first two weeks) had worn down by week 7. Importantly, amastigotes of *Ld22A1*^*−*^ +pXNG4-*22A1* with either PBS or GCV treatment maintained much higher levels of the plasmid (average of 8–28 copies per cell) than *Ld22A1*+/− +pXNG4-*22A1* (average of < 2 copies per cell) (Fig. 6C). Together, these results demonstrated that CYP5122A1 is indispensable for *L. donovani* amastigotes in mice.

### Genetic manipulation of CYP5122A1 altered sterol composition and affected the expression of surface glycoconjugates and promastigote stress responses.

The effects of genetic manipulation of CYP5122A1 on sterol synthesis were analyzed by LC-MS/MS (Table 1). Several 4,14-methylated sterols (lanosterol, 4CH_2_OH-LS, and 4,14-DMZ) were significantly accumulated in *Ld22A1*+/− during log and stationary phases, while CYP5122A1 overexpression caused downregulation of these sterols. In addition, neither FF-MAS nor T-MAS (formed by lanosterol C14-demethylation by CYP51) was detected in the Ld1s WT, *Ld22A1*+/−, *Ld22A1*+/−+pXNG4-*22A1*, or *Ld22A1*^−^ +pXNG4-*22A1* promastigotes. This suggested that lanosterol C4-demethylation, rather than C14-demethylation, appears to be the dominant reaction during ergosterol biosynthesis in these parasites.

**Figure 7.**
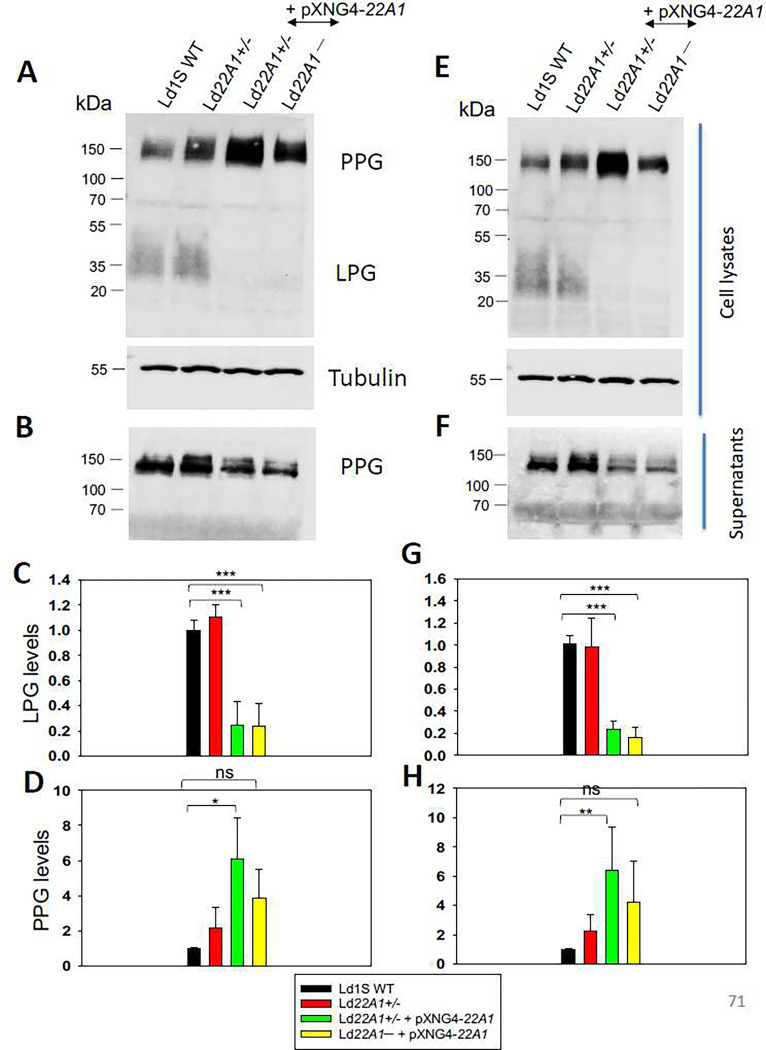
CYP5122A1 overexpression alters the expression of LPG and PPG. Log phase (A-D) and stationary phase (E-H) promastigotes were subjected to Western blot analyses using the monoclonal antibody CA7AE (for LPG and PPG) or an anti-α-tubulin antibody (as loading control). A and E: whole cell lysates. B and F: culture supernatants. Relative expression levels of LPG (C and G) and PPG (D and H) were determined with Ld1S WT as 1.0. Error bars represent standard deviations from three repeats (*: *p* < 0.05, **: *p* < 0.01, ***: *p* < 0.001).

LPG and PPG are two major phosphoglycans in *Leishmania* promastigotes. They are critical for parasite attachment to the midgut of sand flies and for the establishment of infection in mammalian macrophages [44]. LPG is a glycosylphosphatidylinositol (GPI)-anchored cell surface molecule and has been identified as a virulence factor [45]. PPG has the GPI-anchored form present on the cell surface and the secreted form lacking in the GPI anchor [46]. Previously it was found that the levels of GPI-anchored molecules were affected by changes in sterol composition in *Leishmania* [11]. Considering that we observed the altered sterol profiles by genetic manipulation of CYP5122A1, we investigated if the expression of LPG and PPG was also impacted. Western blot analysis (Fig. 7) showed that CYP5122A1 overexpression led to more cellular PPG but less secreted PPG in both log and stationary phases. In addition, the expression of cellular LPG was significantly decreased. Although it was unclear if these changes would have any implications on the parasite infectivity, the results provided more evidence of the association between the sterol composition and the synthesis of important virulence factors in *Leishmania*.

**Figure 8.**
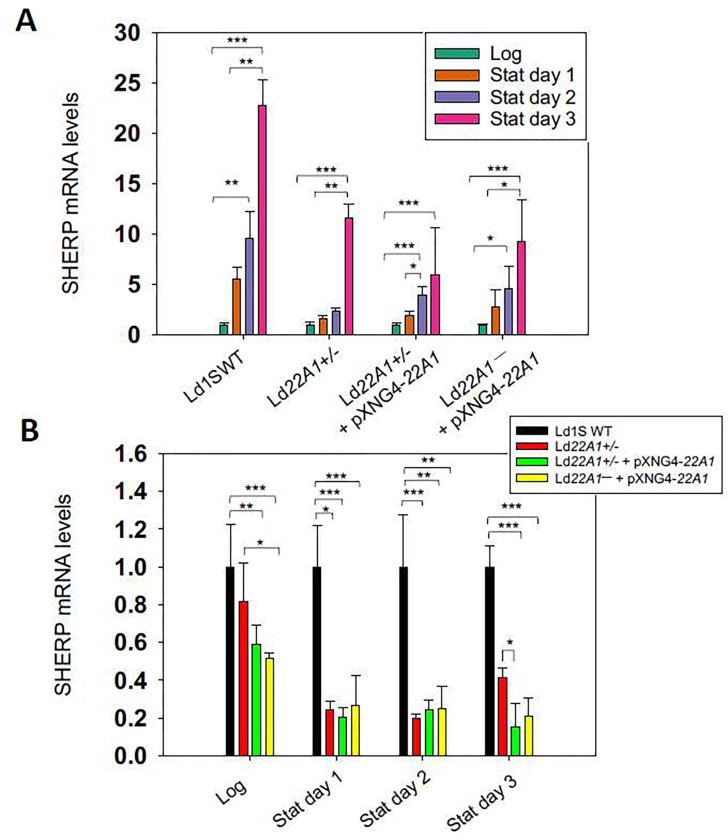
CYP5122A1 half knockout and overexpression reduces the expression of *SHERP*. Total RNA from log phase and stationary phase promastigotes were subjected to RT-qPCR analyses using primers for *SHERP* and 28S rDNA (as loading control). (A) Expression levels of *SHERP* in each parasite line from log phase to stationary phase using log phase levels as 1.0. (B) Expression levels of *SHERP* relative to Ld1s WT (as 1.0). Error bars represent standard deviations from three repeats (*: *p* < 0.05, **: *p* < 0.01, ***: *p* < 0.001).

Next, we examined if genetic manipulation of CYP5122A1 affected the expression of the metacyclic stage-specific marker SHERP (small hydrophilic endoplasmic reticulum-associated protein) [47] (Fig. 8). As expected, *SHERP* mRNA levels were low in Ld1S WT and CYP5122A1 mutants during the log phase. Upon entering the stationary phase, their *SHERP* expression levels were significantly increased. However, the degree of SHERP induction in stationary phase was less pronounced as WT (Fig. 8A). After normalizing the expression levels of *SHERP* in mutants to those in WT, we observed that *CYP5122A1* half knockout and overexpression reduced the *SHERP* expression in both log and stationary phases (Fig. 8B).

**Figure 9.**
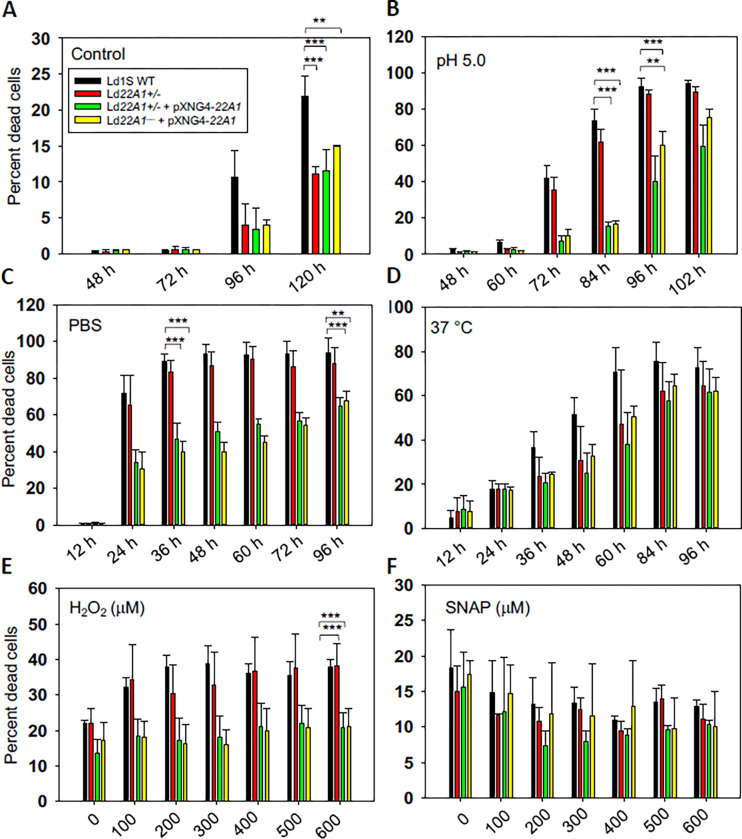
CYP5122A1 overexpression compromises promastigote stress response. (A-D) Day 1 stationary phase promastigotes were incubated under various conditions and percentages of dead cells were determined by flow cytometry at the indicated times. (A) Complete M199 medium, 27 °C, pH7.4 (control). (B) Complete M199 medium, 27 °C, pH 5.0. (C) PBS, 27 °C, pH 7.4. (D) Complete M199 medium, 37 °C, pH 7.4. (E-F) Day 1 stationary phase promastigotes were incubated in various concentrations of H_2_O_2_ (E) or SNAP (F) and percentages of dead cells were determined after 48 hours. Error bars represent standard deviations from three repeats (*: *p* < 0.05, **: *p* < 0.01, ***: *p* < 0.001).

We then assessed the ability of CYP5122A1 mutants to withstand different types of stress in stationary phase. Under the standard culture conditions (complete M199 medium, pH 7.4, 27 °C), less than 1% of dead cells were seen in stationary phase promastigotes after 72 h of incubation (Fig. 9A). However, CYP5122A1 mutants displayed better survival in late stationary phase (6–120 h post inoculation). The acidic pH is one of the environmental stresses imposed on the promastigotes when they reside in the phagolysosome of macrophages. Here, when promastigotes were cultivated at pH 5.0 for 84 h, the percentage of dead cells was less than 20% in CYP5122A1 overexpressors but reached over 60% in Ld1S WT and *Ld22A1*+/− (Fig. 9B). The increased tolerance of *Ld22A1*+/−+pXNG4-*22A1* and *Ld22A1*^−^ +pXNG4-*22A1* to stress was also evident in their responses to starvation. After incubation in PBS in the absence of nutrients for 36 h, they had ~40% of dead cells whereas almost 90% of the WT and *Ld22A1*+/− cells did not survive (Fig. 9C).

When the incubation temperature was set at 37 °C to mimic the mammalian body temperature, the percentage of dead cells increased slower in CYP5122A1 mutants than in Ld1Ss WT although the difference was not statistically significant (Fig. 9D). Apart from physical stresses, *Leishmania* parasites would encounter reactive oxygen and nitrogen species which are produced by macrophages as part of the host defense responses [48]. To evaluate their resistance to oxidative or nitrosative stress (a condition they would encounter as part of the host defense [48]), parasites were incubated in various concentrations of H_2_O_2_ or SNAP (Fig. 9E–F). CYP5122A1 overexpressors had superior viability than WT and *Ld22A1*+/− at H_2_O_2_ concentrations up to 600 μM (*p* < 0.001) but exhibited no difference in response to SNAP. Taken together, CYP5122A1 overexpression rendered the cells more resistant to hostile environment.

### Dual inhibition of CYP5122A1 and CYP51 was required for optimal antileishmanial activities by antifungal azoles.

A panel of twenty marketed antifungal azole drugs was evaluated for their inhibitory activities against *L. donovani* CYP5122A1 and CYP51, along with several other classical antileishmanial agents (Table 2). Results of the inhibition assays showed that all tested antifungal azoles were potent inhibitors of CYP51 (IC_50_s: 0.032 – 0.96 μM) but exhibited much weaker inhibition against CYP5122A1 (IC_50_s ranging from 0.26 to over 100 μM). Specifically, voriconazole and fluconazole marginally inhibited CYP5122A1 at a concentration as high as 100 μM (< 28% and 20% inhibition, respectively). Voriconazole was the most selective inhibitor of CYP51 (>1176-fold over CYP5122A1) and clotrimazole was the least selective one (3.8-fold). DB766, an antileishmanial arylimidamide [49], was identified as a selective inhibitor of CYP5122A1 (>30-fold over CYP51). Two selective CYP51 inhibitors (voriconazole and fluconazole) and two dual CYP51/CYP5122A1 inhibitors (clotrimazole and posaconazole) were further assessed for their binding modes with the two CYP enzymes by UV-Vis spectrophotometric analysis (Supplementary Fig. 7). Clotrimazole exhibited type II binding to both CYP51 and CYP5122A1, indicating that the nitrogen in its imidazole group was directly coordinated with the heme iron at the active site. Posaconazole exhibited type II binding to CYP51, but a type II-like binding to CYP5122A1 (the trough peak at around 410 nm is absent). Fluconazole and voriconazole elicited type II binding with CYP51 but no specific responses with CYP5122A1, consistent with the observation that they were CYP51-selective inhibitors. Moreover, we evaluated the effect of azole antifungal drugs on the proliferation of *L. donovani* LV82 promastigotes (Table 2 and Supplementary Fig. 8). Of these 20 compounds tested, twelve displayed EC_50_ values <10 μM, while another six azoles exhibited EC_50_ values between 10 μM and 25 μM. Interestingly, voriconazole and fluconazole, the weakest inhibitors of CYP5122A1, were the least effective at inhibiting parasite growth with EC_50_ values >100 μM. These results suggested that dual inhibitors of CYP51 and CYP5122A1 were more likely to inhibit *L. donovani* growth.

Sterol analysis of LV82 promastigotes showed that, as expected, both clotrimazole (dual CYP51 and CYP5122A1 inhibitor) and voriconazole (selective CYP51 inhibitor) treatments resulted in the depletion of 4,14-demethylated and 5-dehydro sterols in the downstream pathway of ergosterol biosynthesis, and the accumulation of some 4- and/or 14-methylated sterols (Table 3). However, the extent of change and identities of sterols impacted were different between the two treatments. Lanosterol and 4,14-DMZ, which are the substrates of CYP5122A1 and CYP51, were accumulated to a greater extent in the cells treated with clotrimazole than with voriconazole. Also, 14-methylzymosterol, an intermediate possessing the 14-methyl group but lacking methyl groups at C4, accumulated to an approximately 4-fold greater extent in parasites treated with voriconazole, a selective inhibitor of CYP51, compared to parasites treated with clotrimazole, a compound that inhibits both CYP51 and CYP5122A1. Indeed, the effects of clotrimazole and voriconazole on leishmanial sterol profiles were similar to those observed with posaconazole and fluconazole, respectively [16]. These results showed that, in *L. donovani*, lanosterol undergoes C4-demethylation catalyzed by CYP5122A1 and inhibition of both CYP5122A1- and CYP51-mediated reactions by dual inhibitors like clotrimazole and posaconazole. These dual inhibitors blocked ergosterol biosynthesis at earlier steps with a greater extent and produced better antileishmanial effects than the selective CYP51 inhibitors voriconazole and fluconazole.

### Sensitivity of CYP5122A1 overexpressors to CYP5122A1-selective and dual inhibitors

**Figure 10.**
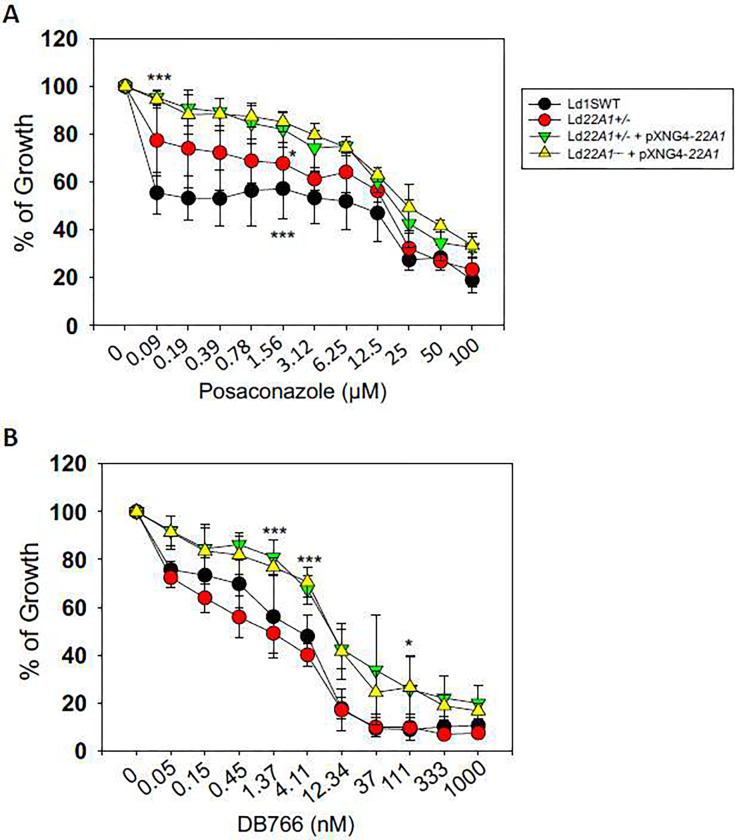
CYP5122A1 overexpression reduces sensitivity to CYP5122A1 inhibitors. Log phase promastigotes were inoculated in various concentrations of posaconazole (A) or DB766 (B) and culture densities were determined after 48 hours. Percentages of growth were indicated relative to control cells grown in the absence of inhibitors. Error bars represent standard deviations from three repeats (*: *p* < 0.05, ***: *p* < 0.001).

To test whether CYP5122A1 overexpression affected the susceptibility to the CYP5122A1-selective and dual inhibitors, we measured the growth of Ld1S WT and CYP5122A1 mutants in the presence or absence of different inhibitors (Fig. 10). Compared to Ld1S WT and *Ld22A1*+/−, CYP5122A1 overexpressors were more resistant to the CYP5122A1-selective inhibitor DB766 (Fig. 19A, EC_50_ = ~12 nM for overexpressors vs 1–4 nM for WT and *Ld22A1*+/−), suggesting that an increased level of CYP5122A1 can bind and buffer the effect of this inhibitor. For the dual inhibitor posaconazole, Ld1S WT showed a bi-phasic response with 0.1 μM being sufficient to reach EC50, while more than 12 μM was needed to inhibit growth further (Fig. 10B). In contrast, CYP5122A1 overexpressors were much more resistant to posaconazole (EC50 = ~25 μM), indicating that increased production of this enzyme can protect parasites against dual inhibitors. An intermediate effect was observed with *Ld22A1*+/−, which suggests compensatory changes in these half knockout parasites.

## Discussion

Sterol C4-demethylation is a vital step in ergosterol/cholesterol biosynthesis (Scheme 1) and this step has been found to differ across biological kingdoms. In vertebrates, plants, and fungi, three enzymes function sequentially to remove the lanosterol C4-methyl groups: a nonheme iron-dependent sterol C4-methyl oxidase (SMO or Erg25), an NAD(P)-dependent 3β-hydroxysteroid dehydrogenase/C4-decarboxylase (3βHD/D or Erg26), and an NADPH-dependent 3-ketosteroid reductase (3-SR or Erg27) [17–19]. In some myxobacteria like *Methylococcus capsulatus*, sterol C4-demethylation is catalyzed by two enzymes that are phylogenetically and biochemically distinct from those in eukaryotes: SdmA (acting as SMO) and SdmB (may affect both decarboxylative oxidation and ketoreduction at C-3) [50]. Animals and yeast only have one sterol C4-methyl oxidase which is involved in the iterative removal of the two C4-methyl groups from sterols [17, 51]. First, sterol C4-methyl oxidase converts the 4,4-dimethylated sterol substrate to a 4α-carboxylate metabolite, which subsequently decarboxylates to remove the first C4-methyl group. To maintain the stereochemical recognition by the enzyme during the removal of the second C4-methyl group, the remaining methyl group at the 4β-(axial) position epimerizes to the more stable 4α-(equatorial) position through the 4-methyl 3-ketosteroid product to re-establish the C4α-methyl configuration. The 3-keto sterol is then reduced by 3-ketosteroid reductase to form the 3β-hydroxy sterol which has the last C4α-methyl group ready for another round of C4-demethylation [52]. To date, the sterol C4-demethylation process in *Leishmania* protozoa was largely unknown and often assumed to be similar to the one in fungi.

In this study, we identified leishmanial CYP5122A1 as a sterol C4-methyl oxidase and unveiled a previously unrecognized important difference in the sterol C4-demethylation process between *Leishmania* and other species. Leishmanial CYP5122A1 was found to be responsible for oxidizing the lanosterol C4-methyl group into first a hydroxyl, then an aldehyde, and finally a carboxylate/formyloxy metabolite. Although the stereochemistry of these metabolites at the C-4 position was not directly ascertained from the NOESY spectrum of the isolated metabolite due to low signal intensity, it can be inferred as a sterol C4-methyl oxidase due to several lines of evidence. First, CYP5122A1 oxidized 4,14-DMZ, which has an α-configuration at both C4- and C14-methyl groups [16], and elicited a type-I binding with 4,14-DMZ (Fig. 2B). It also showed a type I binding specificity to C4-methylated sterols, whereas CYP51 preferred C14-methylated sterols (Fig. 2A). Second, CYP5122A1 converted 4,14-DMZ into a hydroxylated product (peak **15** in Fig. 3B) that is different from the C14-hydroxylmethyl product formed by CYP51 (peak **11** in Fig. 3B), indicating that CYP5122A1 likely acted on the C4-methyl group, rather than the C14-methyl group of 4,14-DMZ. This is further supported by our NMR structural determination of the C4-aldehyde metabolite of lanosterol formed by CYP5122A1 (Fig. 4). Lastly, C4-methylated substrates (lanosterol, 4CH_2_OH-LS, and 4,14-DMZ) were accumulated in *L. donovani* promastigotes when CYP5122A1 was either chemically inhibited (by clotrimazole; Tables 2 and 3) or genetically suppressed (*Ld22A1*+/− heterozygotes; Supplementary Fig. 6 and Table 1).

This is the first report of a CYP-mediated sterol C4-methyl oxidation. Intriguingly, genes encoding CYP5122A1-like proteins were also identified in *T. brucei* and *T. cruzi* which are the causative agents of two important human parasitic diseases, sleeping sickness and Chagas disease, respectively. Their protein sequences are 50 – 53% identical to leishmanial CYP5122A1 [25] and thus belong to the same CYP5122 family. Results from our study could shed light on the roles of these counterparts in *Trypanosoma*. It is still unknown whether trypanosomal CYP5122A1 enzymes possess the same biochemical function or exhibit the same inhibition profile as the leishmanial CYP5122A1 enzyme. Moreover, enzymes that are involved in the C4-demethylation process after C4-oxidation in *Leishmania* (i.e., dehydrogenase/decarboxylase and reductase) remain uncharacterized, let alone other enzymes responsible for the multi-step conversion of lanosterol to ergosterol in these parasites. An ortholog of yeast Erg25 has been found in the *Leishmania* genome but its function remains to be characterized [53]. As such, the sterol biosynthetic pathway in protozoa warrants future dedicated investigation to fully understand the unique sterol biology of these single cell parasites. As demonstrated in this study, it is premature to assume these protozoan parasites behave like fungi or mammalian species.

The essentiality of *CYP5122A1* was first investigated in *L. donovani* promastigotes by using the homologous replacement approach [25]. Deleting both alleles of *CYP5122A1* was found to be lethal, which suggested that the gene is required for parasite survival. However, failure to obtain the null mutant in this way is often considered weak evidence for gene essentiality [54]. In the present study, the more rigorous forced plasmid shuffle approach was applied to examine the gene essentiality. We were able to generate the null mutant transfected with the plasmid containing *CYP5122A1* and a negative selection mark (*TK*). The null mutant without the episomal *CYP5122A1* could not survive. In the presence of negative selection, the parasites lacking in the plasmid would be favored, yet retention of the plasmid was observed in parasites after multiple passages. This provided compelling evidence supporting the essentiality of *CYP5122A1* in *L. donovani* promastigotes. Moreover, a more comprehensive assessment should also take into account the amastigote stage of the parasite life cycle. Unlike promastigotes, intracellular amastigotes live in a semi-quiescent state characterized by a negligible proliferation rate, a lower biosynthetic capacity, a reduced bio-energetic level, and a stringent metabolic response [55, 56]. In particular, amastigotes acquire most of the sterols by salvage from the host although they retain the capacity of de novo synthesis [57]. It was observed that cholesterol was much more abundant than ergostane-based sterols in amastigotes [11]. In view of such different sterol profiles during the *Leishmania* life cycle, we investigated whether CYP5122A1 was still essential during the amastigote stage using a mouse model of visceral leishmaniasis infection. Under the pressure of negative selection, the null mutant with the episomal complementation of *CYP5122A1* was able to proliferate in mice and the plasmid was highly retained. These results demonstrate the essentiality of *CYP5122A1* in *L. donovani* amastigotes, which is critical for the genetic validation of CYP5122A1 as a drug target.

Antifungal azole drugs have been assessed for their potential to be used as antileishmanial agents. Studies showed that they were potent inhibitors of leishmanial CYP51 that can block ergosterol biosynthesis, but it was not fully understood why they had vastly different antileishmanial effects *in vitro* [13, 14, 58]. In this study, we screened a panel of twenty marketed antifungal azole drugs for the inhibition of CYP51 and CYP5122A1 using a fluorescence-based inhibition assay. Based on the selectivity towards the CYP enzymes, they were grouped into CYP51-selective inhibitors (e.g., fluconazole and voriconazole) and dual inhibitors of both CYP enzymes (e.g., posaconazole and clotrimazole). Overall, dual inhibitors exhibited higher antileishmanial activities against LV82 promastigotes than CYP51-selective inhibitors. This supports the critical role of CYP5122A1 in determining the azole activities against the parasites. When designing new compounds that target the sterol biosynthesis in *Leishmania*, inhibiting both CYP51 and CYP5122A1 may be a better option than inhibiting CYP51 alone. A previous study on a potent antileishmanial arylimidamide DB766 implicated CYP5122A1 in the antileishmanial action of the compound [26], which could be supported by our finding that DB766 is a strong CYP5122A1 inhibitor with IC_50_ of 1.0 ± 0.1 μM, although additional mechanisms are likely involved to account for the potent antileishmanial activity of the compound with IC_50_ values ranging from 0.014 to 0.5 μM against multiple *Leishmania* species [49] and against wild type *L. donovani* strain 1S2D (0.004 μM; Fig. 10A).

When analyzing the sterol profiles of azole-treated promastigotes along with our previous findings [16], we found that CYP51-selective inhibitors and dual inhibitors resulted in distinct patterns of sterol accumulation. The underlying cause could be the branched ergosterol biosynthesis pathway that we previously proposed [16]. Lanosterol, the substrate of both CYP51 and CYP5122A1, can undergo either a C4- or C14-demethylation reaction. Dual inhibitors caused lanosterol accumulation by inhibiting both reactions whereas CYP51-selective inhibitors would allow C4-demethylation to proceed. Furthermore, sterol analysis of genetic mutants showed accumulation of 4,14-methylated sterols (lanosterol, 4CH_2_OH-LS, and 4,14-DMZ) in *Ld22A1*+/− and downregulation in CYP5122A1 overexpressors (*Ld22A1*+/−+pXNG4-22A1 and *Ld22A1*^−^+pXNG4-22A1), suggesting that C4-demethylation, rather than C14-demethylation, is the dominant reaction for lanosterol metabolism in *Leishmania*. This is further supported by the lack of detection of FF-MAS and T-MAS in the *Leishmania* parasites (Tables 1 and 3), which were expected to be formed by the lanosterol C14-demethylation catalyzed by CYP51.

There are several possible reasons for CYP5122A1 being essential to both *L. donovani* promastigotes and amastigotes. First, ergostane-based sterols are crucial and host-derived cholesterol is insufficient for maintaining the cell membrane rigidity. Second, the accumulation of 4,14-methylated sterols (e.g., lanosterol) and 4-methylated sterols have detrimental effects on *Leishmania* parasites. It has been found that the lipid domain/raft formation in the biological membranes is dependent on the sterol component having a structure that allows tight packing with lipids [59]. Ergosterol is conformationally and dynamically more restricted than cholesterol and has a higher tendency to promote lipid domain formation. Lanosterol is the bulkiest one among these three and is the least effective in inducing lipid packing [60, 61]. Therefore, changes in sterol compositions could affect the lipid domains which have been implicated in numerous cellular processes. It was reported that the accumulation of C4-methyl sterols might serve as a signal for low oxygen and cell stress in fission yeast [62]. Similarly, C4-methyl sterols were also important for stress and hypoxia adaption in *Aspergillus fumigatus* [63]. However, whether C4-methyl sterols have biological impacts on *Leishmania* parasites is unclear and requires further investigation.

When we investigated the effects of CYP5122A1 overexpression, lower levels of *SHERP* expression were observed in CYP5122A1 overexpressors relative to Ld1S WT in both log and stationary phases. SHERP, expressed predominantly in metacyclic parasites, is required for *Leishmania* metacyclogenesis in sand flies [64, 65]. Studies suggested that it may function in modulating cellular processes related to membrane organization and/or acidification [66, 67]. Reduction of SHERP expression indicated the differentiation defects of CYP5122A1 overexpressors. In addition, they exhibited delayed growth, different expression pattern for LPG/PPG, and altered stress responses (Figs. 7 and 9; and Supplemental Fig. 6A). Given that CYP5122A1 overexpression did not impact the bulk sterol composition but caused the depletion of 4-methylated sterols, it is likely that the low levels of these intermediates serve as a signal to suppress differentiation. Finally, while dual inhibitors like DB766 and posaconazole exhibited potent anti-proliferation effect, CYP5122A1 overexpression conferred significant protection (Fig. 10), which reinforced the importance of sterol C4-demethylation in *Leishmania*.

## Conclusions

In summary, our study elucidated that CYP5122A1 is a sterol C4-methyl oxidase involved in the sterol C4-demethylation process in *Leishmania*. The essentiality of CYP5122A1 was verified in both *L. donovani* promastigotes and intracellular amastigotes. Overexpression of CYP5122A1 resulted in growth delay, differentiation defects, increased tolerance to environmental stresses, and altered expression of surface glycoconjugates. Antifungal azoles acting as dual inhibitors of CYP51 and CYP5122A1 had higher antileishmanial activity against *L. donovani* promastigotes than CYP51-selective inhibitors, suggesting a new strategy to develop therapeutic agents to target the sterol biosynthetic pathway in *Leishmania*.

## References

[1] Bates PA. Revising Leishmania’s life cycle. Nat Microbiol. 2018;3(5):529–30. doi: 10.1038/s41564-018-0154-2. 29693656

[2] Kima PE. The amastigote forms of Leishmania are experts at exploiting host cell processes to establish infection and persist. Int J Parasitol. 2007;37(10):1087–96. Epub 20070429. doi: 10.1016/j.ijpara.2007.04.007. 17543969 PMC2043126

[3] Serafim TD, Coutinho-Abreu IV, Oliveira F, Meneses C, Kamhawi S, Valenzuela JG. Sequential blood meals promote Leishmania replication and reverse metacyclogenesis augmenting vector infectivity. Nat Microbiol. 2018;3(5):548–55. Epub 20180319. doi: 10.1038/s41564-018-0125-7. 29556108 PMC6007031

[4] World Health Organization. [cited 2023 June 25]. Available from: https://www.who.int/news-room/fact-sheets/detail/leishmaniasis.

[5] Chappuis F, Sundar S, Hailu A, Ghalib H, Rijal S, Peeling RW, Visceral leishmaniasis: what are the needs for diagnosis, treatment and control? Nat Rev Microbiol. 2007;5(11):873–82. Epub 2007/10/17. doi: nrmicro1748 [pii] 10.1038/nrmicro1748. 17938629

[6] Karmakar S, Ismail N, Oliveira F, Oristian J, Zhang WW, Kaviraj S, Preclinical validation of a live attenuated dermotropic Leishmania vaccine against vector transmitted fatal visceral leishmaniasis. Commun Biol. 2021;4(1):929. Epub 20210730. doi: 10.1038/s42003-021-02446-x. 34330999 PMC8324786

[7] Alves F, Bilbe G, Blesson S, Goyal V, Monnerat S, Mowbray C, Recent development of visceral leishmaniasis treatments: successes, pitfalls, and perspectives. Clin Microbiol Rev. 2018;31(4). Epub 20180829. doi: 10.1128/CMR.00048-18. PMC6148188 30158301

[8] Sundar S, Singh B. Emerging therapeutic targets for treatment of leishmaniasis. Expert Opin Ther Targets. 2018;22(6):467–86. Epub 20180509. doi: 10.1080/14728222.2018.1472241. 29718739 PMC6047532

[9] de Souza W, Rodrigues JC. Sterol biosynthesis pathway as target for anti-trypanosomatid drugs. Interdiscip Perspect Infect Dis. 2009;2009:642502. Epub 20090805. doi: 10.1155/2009/642502. 19680554 PMC2721973

[10] Yao C, Gaur Dixit U, Barker JH, Teesch LM, Love-Homan L, Donelson JE, Attenuation of Leishmania infantum chagasi metacyclic promastigotes by sterol depletion. Infect Immun. 2013;81(7):2507–17. Epub 20130429. doi: 10.1128/IAI.00214-13. 23630964 PMC3697599

[11] Xu W, Hsu FF, Baykal E, Huang J, Zhang K. Sterol biosynthesis is required for heat resistance but not extracellular survival in leishmania. PLoS pathogens. 2014;10(10):e1004427. Epub 20141023. doi: 10.1371/journal.ppat.1004427. 25340392 PMC4207814

[12] Mukherjee S, Moitra S, Xu W, Hernandez V, Zhang K. Sterol 14-alpha-demethylase is vital for mitochondrial functions and stress tolerance in Leishmania major. PLoS Pathog. 2020;16(8):e1008810. Epub 20200820. doi: 10.1371/journal.ppat.1008810. 32817704 PMC7462297

[13] Beach DH, Goad LJ, Holz GG Jr., Effects of antimycotic azoles on growth and sterol biosynthesis of Leishmania promastigotes. Mol Biochem Parasitol. 1988;31(2):149–62. 2847043 10.1016/0166-6851(88)90166-1

[14] Yamamoto ES, Jesus JA, Bezerra-Souza A, Laurenti MD, Ribeiro SP, Passero LFD. Activity of fenticonazole, tioconazole and nystatin on New World Leishmania species. Curr Top Med Chem. 2018;18(27):2338–46. doi: 10.2174/1568026619666181220114627. 30569856

[15] Buckner FS, Wilson AJ. Colorimetric assay for screening compounds against Leishmania amastigotes grown in macrophages. Am J Trop Med Hyg. 2005;72(5):600–5. Epub 2005/05/14. doi: 72/5/600 [pii]. 15891135

[16] Feng M, Jin Y, Yang S, Joachim AM, Ning Y, Mori-Quiroz LM, Sterol profiling of Leishmania parasites using a new HPLC-tandem mass spectrometry-based method and antifungal azoles as chemical probes reveals a key intermediate sterol that supports a branched ergosterol biosynthetic pathway. Int J Parasitol Drugs Drug Resist. 2022;20:27–42. Epub 20220815. doi: 10.1016/j.ijpddr.2022.07.003. 35994895 PMC9418051

[17] Bard M, Bruner DA, Pierson CA, Lees ND, Biermann B, Frye L, Cloning and characterization of ERG25, the Saccharomyces cerevisiae gene encoding C-4 sterol methyl oxidase. Proc Natl Acad Sci U S A. 1996;93(1):186–90. doi: 10.1073/pnas.93.1.186. 8552601 PMC40203

[18] Gachotte D, Barbuch R, Gaylor J, Nickel E, Bard M. Characterization of the Saccharomyces cerevisiae ERG26 gene encoding the C-3 sterol dehydrogenase (C-4 decarboxylase) involved in sterol biosynthesis. Proc Natl Acad Sci U S A. 1998;95(23):13794–9. doi: 10.1073/pnas.95.23.13794. 9811880 PMC24900

[19] Gachotte D, Sen SE, Eckstein J, Barbuch R, Krieger M, Ray BD, Characterization of the Saccharomyces cerevisiae ERG27 gene encoding the 3-keto reductase involved in C-4 sterol demethylation. Proc Natl Acad Sci U S A. 1999;96(22):12655–60. doi: 10.1073/pnas.96.22.12655. 10535978 PMC23033

[20] Rahier A. Dissecting the sterol C-4 demethylation process in higher plants. From structures and genes to catalytic mechanism. Steroids. 2011;76(4):340–52. Epub 20101213. doi: 10.1016/j.steroids.2010.11.011. 21147141

[21] Lepesheva GI, Waterman MR. Sterol 14alpha-demethylase (CYP51) as a therapeutic target for human trypanosomiasis and leishmaniasis. Curr Top Med Chem. 2011;11(16):2060–71. 21619513 10.2174/156802611796575902 PMC3391166

[22] Hargrove TY, Wawrzak Z, Liu J, Nes WD, Waterman MR, Lepesheva GI. Substrate preferences and catalytic parameters determined by structural characteristics of sterol 14alpha-demethylase (CYP51) from Leishmania infantum. J Biol Chem. 2011;286(30):26838–48. doi: 10.1074/jbc.M111.237099. 21632531 PMC3143644

[23] Friggeri L, Hargrove TY, Rachakonda G, Blobaum AL, Fisher P, de Oliveira GM, Sterol 14alpha-demethylase structure-based optimization of drug candidates for human infections with the protozoan Trypanosomatidae. J Med Chem. 2018;61(23):10910–21. Epub 20181130. doi: 10.1021/acs.jmedchem.8b01671. 30451500 PMC6467724

[24] da Silva Santos-Junior PF, Schmitt M, de Araujo-Junior JX, da Silva-Junior EF. Sterol 14alpha-demethylase from Trypanosomatidae parasites as a promising target for designing new antiparasitic agents. Curr Top Med Chem. 2021;21(21):1900–21. doi: 10.2174/1568026621666210303144448. 33655860

[25] Verma S, Mehta A, Shaha C. CYP5122A1, a novel cytochrome P450 is essential for survival of Leishmania donovani. PloS one. 2011;6(9):e25273. Epub 2011/10/04. doi: 10.1371/journal.pone.0025273. 21966477 PMC3179497

[26] Pandharkar T, Zhu X, Mathur R, Jiang J, Schmittgen TD, Shaha C, Studies on the antileishmanial mechanism of action of the arylimidamide DB766: azole interactions and role of CYP5122A1. Antimicrob Agents Chemother. 2014;58(8):4682–9. Epub 2014/06/04. doi: 10.1128/AAC.02405-14. 24890590 PMC4135980

[27] Lepesheva GI, Park HW, Hargrove TY, Vanhollebeke B, Wawrzak Z, Harp JM, Crystal structures of Trypanosoma brucei sterol 14alpha-demethylase and implications for selective treatment of human infections. J Biol Chem. 2010;285(3):1773–80. doi: 10.1074/jbc.M109.067470. 19923211 PMC2804335

[28] Abdelhameed A, Feng M, Joice AC, Zywot EM, Jin Y, La Rosa C, Synthesis and Antileishmanial Evaluation of Arylimidamide-Azole Hybrids Containing a Phenoxyalkyl Linker. ACS Infect Dis. 2021;7(7):1901–22. Epub 20210204. doi: 10.1021/acsinfecdis.0c00855. 33538576 PMC8553517

[29] de Ibarra AA, Howard JG, Snary D. Monoclonal antibodies to Leishmania tropica major: specificities and antigen location. Parasitology. 1982;85 (Pt 3):523–31. doi: 10.1017/s0031182000056304. 6184664

[30] Schenkman JB, Jansson I. Spectral analyses of cytochromes P450. Methods Mol Biol. 2006;320:11–8. doi: 10.1385/1-59259-998-2:11. 16719370

[31] Kapler GM, Coburn CM, Beverley SM. Stable transfection of the human parasite Leishmania major delineates a 30-kilobase region sufficient for extrachromosomal replication and expression. Mol Cell Biol. 1990;10(3):1084–94. doi: 10.1128/mcb.10.3.1084-1094.1990. 2304458 PMC360971

[32] Moitra S, Basu S, Pawlowic M, Hsu FF, Zhang K. De novo synthesis of phosphatidylcholine is essential for the promastigote but not amastigote stage in Leishmania major. Front Cell Infect Microbiol. 2021;11:647870. Epub 20210312. doi: 10.3389/fcimb.2021.647870. 33777852 PMC7996062

[33] Murta SM, Vickers TJ, Scott DA, Beverley SM. Methylene tetrahydrofolate dehydrogenase/cyclohydrolase and the synthesis of 10-CHO-THF are essential in Leishmania major. Molecular microbiology. 2009;71(6):1386–401. Epub 2009/02/03. doi: 10.1111/j.1365-2958.2009.06610.x. 19183277 PMC2692627

[34] Titus RG, Marchand M, Boon T, Louis JA. A limiting dilution assay for quantifying Leishmania major in tissues of infected mice. Parasite Immunol. 1985;7(5):545–55. doi: 10.1111/j.1365-3024.1985.tb00098.x. 3877902

[35] Livak KJ, Schmittgen TD. Analysis of relative gene expression data using real-time quantitative PCR and the 2(-Delta Delta C(T)) Method. Methods. 2001;25(4):402–8. doi: 10.1006/meth.2001.1262. 11846609

[36] Joice AC, Yang S, Farahat AA, Meeds H, Feng M, Li J, Antileishmanial Efficacy and Pharmacokinetics of DB766-Azole Combinations. Antimicrob Agents Chemother. 2018;62(1). Epub 20171221. doi: 10.1128/AAC.01129-17. PMC5740383 29061761

[37] Ritz C, Baty F, Streibig JC, Gerhard D. Dose-response analysis using R. PLoS One. 2015;10(12):e0146021. Epub 20151230. doi: 10.1371/journal.pone.0146021. 26717316 PMC4696819

[38] Lepesheva GI, Nes WD, Zhou W, Hill GC, Waterman MR. CYP51 from Trypanosoma brucei is obtusifoliol-specific. Biochemistry. 2004;43(33):10789–99. doi: 10.1021/bi048967t. 15311940

[39] Lepesheva GI, Zaitseva NG, Nes WD, Zhou W, Arase M, Liu J, CYP51 from Trypanosoma cruzi: a phyla-specific residue in the B’ helix defines substrate preferences of sterol 14alpha-demethylase. J Biol Chem. 2006;281(6):3577–85. doi: 10.1074/jbc.M510317200. 16321980

[40] Schenkman JB, Cinti DL, Orrenius S, Moldeus P, Kraschnitz R. The nature of the reverse type I (modified type II) spectral change in liver microsomes. Biochemistry. 1972;11(23):4243–51. Epub 1972/11/07. doi: 10.1021/bi00773a008. 5079897

[41] Mast N, Zheng W, Stout CD, Pikuleva IA. Binding of a cyano- and fluoro-containing drug bicalutamide to cytochrome P450 46A1: unusual features and spectral response. J Biol Chem. 2013;288(7):4613–24. Epub 20130103. doi: 10.1074/jbc.M112.438754. 23288837 PMC3576067

[42] Fischer RT, Trzaskos JM, Magolda RL, Ko SS, Brosz CS, Larsen B. Lanosterol 14 alpha-methyl demethylase. Isolation and characterization of the third metabolically generated oxidative demethylation intermediate. J Biol Chem. 1991;266(10):6124–32. 2007571

[43] Dacher M, Morales MA, Pescher P, Leclercq O, Rachidi N, Prina E, Probing druggability and biological function of essential proteins in Leishmania combining facilitated null mutant and plasmid shuffle analyses. Molecular microbiology. 2014;93(1):146–66. doi: 10.1111/mmi.12648. 24823804

[44] Valente M, Castillo-Acosta VM, Vidal AE, Gonzalez-Pacanowska D. Overview of the role of kinetoplastid surface carbohydrates in infection and host cell invasion: prospects for therapeutic intervention. Parasitology. 2019;146(14):1743–54. Epub 20191011. doi: 10.1017/S0031182019001355. 31603063 PMC6939169

[45] Spath GF, Epstein L, Leader B, Singer SM, Avila HA, Turco SJ, Lipophosphoglycan is a virulence factor distinct from related glycoconjugates in the protozoan parasite Leishmania major. Proc Natl Acad Sci U S A. 2000;97(16):9258–63. doi: 10.1073/pnas.160257897. 10908670 PMC16855

[46] Borges AR, Link F, Engstler M, Jones NG. The glycosylphosphatidylinositol anchor: a linchpin for cell surface versatility of trypanosomatids. Front Cell Dev Biol. 2021;9:720536. Epub 20211101. doi: 10.3389/fcell.2021.720536. 34790656 PMC8591177

[47] Coulson RM, Smith DF. Isolation of genes showing increased or unique expression in the infective promastigotes of Leishmania major. Mol Biochem Parasitol. 1990;40(1):63–75. doi: 10.1016/0166-6851(90)90080-6. 2348831

[48] Van Assche T, Deschacht M, da Luz RA, Maes L, Cos P. Leishmania-macrophage interactions: insights into the redox biology. Free Radic Biol Med. 2011;51(2):337–51. Epub 20110514. doi: 10.1016/j.freeradbiomed.2011.05.011. 21620959

[49] Wang MZ, Zhu X, Srivastava A, Liu Q, Sweat JM, Pandharkar T, Novel arylimidamides for treatment of visceral leishmaniasis. Antimicrob Agents Chemother. 2010;54(6):2507–16. Epub 20100405. doi: 10.1128/AAC.00250-10. 20368397 PMC2876428

[50] Lee AK, Banta AB, Wei JH, Kiemle DJ, Feng J, Giner JL, C-4 sterol demethylation enzymes distinguish bacterial and eukaryotic sterol synthesis. Proc Natl Acad Sci U S A. 2018;115(23):5884–9. Epub 20180521. doi: 10.1073/pnas.1802930115. 29784781 PMC6003346

[51] He M, Kratz LE, Michel JJ, Vallejo AN, Ferris L, Kelley RI, Mutations in the human SC4MOL gene encoding a methyl sterol oxidase cause psoriasiform dermatitis, microcephaly, and developmental delay. J Clin Invest. 2011;121(3):976–84. doi: 10.1172/JCI42650. 21285510 PMC3049385

[52] Nes WD. Biosynthesis of cholesterol and other sterols. Chemical reviews. 2011;111(10):6423–51. Epub 20110908. doi: 10.1021/cr200021m. 21902244 PMC3191736

[53] Cosentino RO, Aguero F. Genetic profiling of the isoprenoid and sterol biosynthesis pathway genes of Trypanosoma cruzi. PLoS One. 2014;9(5):e96762. Epub 20140514. doi: 10.1371/journal.pone.0096762. 24828104 PMC4020770

[54] Jones NG, Catta-Preta CMC, Lima A, Mottram JC. Genetically Validated Drug Targets in Leishmania: Current Knowledge and Future Prospects. ACS Infect Dis. 2018;4(4):467–77. Epub 2018/02/01. doi: 10.1021/acsinfecdis.7b00244. 29384366 PMC5902788

[55] Saunders EC, Ng WW, Kloehn J, Chambers JM, Ng M, McConville MJ. Induction of a stringent metabolic response in intracellular stages of Leishmania mexicana leads to increased dependence on mitochondrial metabolism. PLoS Pathog. 2014;10(1):e1003888. Epub 20140123. doi: 10.1371/journal.ppat.1003888. 24465208 PMC3900632

[56] Jara M, Berg M, Caljon G, de Muylder G, Cuypers B, Castillo D, Macromolecular biosynthetic parameters and metabolic profile in different life stages of Leishmania braziliensis: Amastigotes as a functionally less active stage. PLoS One. 2017;12(7):e0180532. Epub 20170725. doi: 10.1371/journal.pone.0180532. 28742826 PMC5526552

[57] Zhang K. Balancing de novo synthesis and salvage of lipids by Leishmania amastigotes. Curr Opin Microbiol. 2021;63:98–103. Epub 20210723. doi: 10.1016/j.mib.2021.07.004. 34311265 PMC8463422

[58] Emami S, Tavangar P, Keighobadi M. An overview of azoles targeting sterol 14alpha-demethylase for antileishmanial therapy. Eur J Med Chem. 2017;135:241–59. Epub 20170421. doi: 10.1016/j.ejmech.2017.04.044. 28456033

[59] Xu X, London E. The effect of sterol structure on membrane lipid domains reveals how cholesterol can induce lipid domain formation. Biochemistry. 2000;39(5):843–9. doi: 10.1021/bi992543v. 10653627

[60] Xu X, Bittman R, Duportail G, Heissler D, Vilcheze C, London E. Effect of the structure of natural sterols and sphingolipids on the formation of ordered sphingolipid/sterol domains (rafts). Comparison of cholesterol to plant, fungal, and disease-associated sterols and comparison of sphingomyelin, cerebrosides, and ceramide. J Biol Chem. 2001;276(36):33540–6. Epub 20010629. doi: 10.1074/jbc.M104776200. 11432870

[61] Cournia Z, Ullmann GM, Smith JC. Differential effects of cholesterol, ergosterol and lanosterol on a dipalmitoyl phosphatidylcholine membrane: a molecular dynamics simulation study. J Phys Chem B. 2007;111(7):1786–801. Epub 20070130. doi: 10.1021/jp065172i. 17261058

[62] Hughes AL, Lee CY, Bien CM, Espenshade PJ. 4-Methyl sterols regulate fission yeast SREBP-Scap under low oxygen and cell stress. J Biol Chem. 2007;282(33):24388–96. Epub 20070626. doi: 10.1074/jbc.M701326200. 17595166 PMC2262954

[63] Blosser SJ, Merriman B, Grahl N, Chung D, Cramer RA. Two C4-sterol methyl oxidases (Erg25) catalyse ergosterol intermediate demethylation and impact environmental stress adaptation in Aspergillus fumigatus. Microbiology (Reading). 2014;160(Pt 11):2492–506. Epub 20140808. doi: 10.1099/mic.0.080440-0. 25107308 PMC4219106

[64] Doehl JS, Sadlova J, Aslan H, Pruzinova K, Metangmo S, Votypka J, Leishmania HASP and SHERP genes are required for in vivo differentiation, parasite transmission and virulence attenuation in the host. PLoS Pathog. 2017;13(1):e1006130. Epub 20170117. doi: 10.1371/journal.ppat.1006130. 28095465 PMC5271408

[65] Sadlova J, Price HP, Smith BA, Votypka J, Volf P, Smith DF. The stage-regulated HASPB and SHERP proteins are essential for differentiation of the protozoan parasite Leishmania major in its sand fly vector, Phlebotomus papatasi. Cell Microbiol. 2010;12(12):1765–79. doi: 10.1111/j.1462-5822.2010.01507.x. 20636473 PMC3015063

[66] Knuepfer E, Stierhof YD, McKean PG, Smith DF. Characterization of a differentially expressed protein that shows an unusual localization to intracellular membranes in Leishmania major. Biochem J. 2001;356(Pt 2):335–44. doi: 10.1042/0264-6021:3560335. 11368759 PMC1221843

[67] Moore B, Miles AJ, Guerra-Giraldez C, Simpson P, Iwata M, Wallace BA, Structural basis of molecular recognition of the Leishmania small hydrophilic endoplasmic reticulum-associated protein (SHERP) at membrane surfaces. J Biol Chem. 2011;286(11):9246–56. Epub 20101124. doi: 10.1074/jbc.M110.130427. 21106528 PMC3059043

